# Impact of oral metronidazole treatment on the vaginal microbiota and correlates of treatment failure

**DOI:** 10.1016/j.ajog.2019.08.008

**Published:** 2020-02

**Authors:** Marijn C. Verwijs, Stephen K. Agaba, Alistair C. Darby, Janneke H.H. M. van de Wijgert

**Affiliations:** a Institute of Infection and Global Health, University of Liverpool, Liverpool, United Kingdom; b Rinda Ubuzima, Kigali, Rwanda; c Centre for Genomic Research, University of Liverpool, Liverpool, United Kingdom; d Julius Center for Health Sciences and Primary Care, University Medical Center Utrecht, Utrecht University, Utrecht, The Netherlands

**Keywords:** 16S rRNA gene sequencing, anaerobes, antibiotics, bacterial vaginosis, biofilm, *Lactobacilli*, metronidazole, trichomoniasis, vaginal dysbiosis, vaginal microbiota

## Abstract

**Background:**

Metronidazole is the first-line treatment for bacterial vaginosis, but cure rates are suboptimal and recurrence rates high.

**Objectives:**

To evaluate the impact of a standard course of oral metronidazole treatment (500 mg twice per day for 7 days) on the vaginal microbiota of Rwandan bacterial vaginosis patients using microscopy and 16S rRNA gene sequencing, and to evaluate correlates of treatment failure.

**Study Design:**

HIV-negative, nonpregnant women aged 18–45 years with bacterial vaginosis and/or *Trichomonas vaginalis* (N=68) were interviewed and sampled before and after metronidazole treatment. They were also screened, and treated if applicable, for other urogenital infections. The vaginal microbiota was assessed by Gram stain Nugent scoring, Illumina 16S rRNA HiSeq sequencing (relative abundances), and BactQuant 16S gene quantitative polymerase chain reaction (estimated concentrations). Only women with a pretreatment Nugent score of 7–10 and a valid posttreatment Nugent score (N=55) were included in metronidazole treatment failure analyses, with treatment failure defined as a posttreatment Nugent score of 4–10.

**Results:**

The bacterial vaginosis cure rate by Nugent scoring was 54.5%. The mean total vaginal bacterial concentration declined from 6.59 to 5.85 log_10_/μL (*P*<.001), which was mostly due to a reduction in mean bacterial vaginosis-associated anaerobes concentration (all bacterial vaginosis-associated anaerobe taxa combined) from 6.23 to 4.55 log_10_/μL (*P*<.001). However, only 16.4% of women had a bacterial vaginosis anaerobes concentration reduction of more than 50%, and only 3 women had complete eradication. The mean concentration of lactobacilli (all species combined) increased from 4.98 to 5.56 log_10_/μL (*P*=.017), with *L. iners* being the most common species pre- and posttreatment. The mean concentration of pathobionts (defined as Proteobacteria, streptococci, staphylococci, enterococci, and a few others) did not change significantly: from 1.92 log_10_/μL pretreatment to 2.01 log_10_/μL posttreatment (*P*=.939). Pretreatment pathobionts concentration, and having a pretreatment vaginal microbiota type containing more than 50% *Gardnerella vaginalis* (compared with less than 50%), were associated with increased likelihood of treatment failure, but the latter did not reach statistical significance (*P*=.044 and *P*=.084, respectively).

**Conclusions:**

Metronidazole alone may not cure women with high *G. vaginalis* relative abundance, potentially due to biofilm presence, and women with high pathobionts concentration. These women may benefit from additional biofilm-disrupting and/or pathobiont-targeting treatments.

Click Supplemental Materials under article title in Contents at **ajog.org**

Most women have an optimal vaginal microbiota (VMB) dominated by lactobacilli, but vaginal dysbiosis is highly prevalent.^1^ The most common type of vaginal dysbiosis is bacterial vaginosis (BV), which is characterized by a reduction of lactobacilli and an increase of other anaerobes, usually leading to increased species diversity.^2^, ^3^ Some women carry microorganisms that do not necessarily dominate the VMB but have a greater pathogenic potential than BV anaerobes, such as bacterial pathobionts (including most Proteobacteria, streptococci, staphylococci, and enterococci), *Candida albicans* (which is the main cause of vulvovaginal candidiasis), or *Trichomonas vaginalis*.^2^, ^3^

**AJOG at a Glance**

**Why was this study conducted?**

To evaluate the impact of 7-day oral metronidazole treatment on the vaginal microbiota of Rwandan bacterial vaginosis patients using microscopy and 16S rRNA gene sequencing, and correlates of treatment failure.

**Key findings**

Metronidazole treatment decreased bacterial vaginosis-associated anaerobes, but not by as much as expected, and increased lactobacilli. Pathobionts (bacteria typically associated with hospital infections and neonatal sepsis) were not affected. Treatment failure was associated with high levels of pretreatment *Gardnerella vaginalis* or pathobionts.

**What does this add to what is known?**

The sequencing data explain why metronidazole treatment is associated with suboptimal cure and high recurrence rates. Women with high pretreatment *Gardnerella* may need biofilm-disrupting treatment, and women with high pretreatment pathobionts may need additional antibiotics.

Vaginal dysbiosis can cause symptoms but is often asymptomatic.^2^, ^3^ Both symptomatic and asymptomatic vaginal dysbiosis have been associated with pelvic inflammatory disease, HIV acquisition, and adverse pregnancy outcomes.^3^, ^4^, ^5^, ^6^ Symptomatic dysbiosis is most commonly diagnosed empirically or syndromically without laboratory testing.^7^ However, in research settings, BV is diagnosed by Nugent scoring of vaginal Gram stains or by the Amsel criteria.^8^, ^9^ BV is treated with antibiotics, of which oral and vaginal formulations of metronidazole or clindamycin are most commonly used.^10^ Although short-term BV cure rates of multiday oral and vaginal metronidazole regimens are 65%–90%,^11^, ^12^ recurrence rates are high.^13^, ^14^

Metronidazole is a nitroimidazole-class drug. Under anaerobic conditions, anaerobic bacteria and some parasites (including *T. vaginalis*) metabolize metronidazole into nitroso radicals, which break microbial DNA and cause cell lysis.^15^, ^16^ Culture studies have shown that lactobacilli are not sensitive to metronidazole.^17^ Metronidazole has thus far been associated with low levels of antimicrobial resistance.^18^ The high recurrence rate is therefore often hypothesized to be due to vaginal mucosal biofilm formation by *G. vaginalis* and other BV-associated anaerobes but this has not been confirmed.^19^

We investigated the impact of the most commonly used oral metronidazole treatment regimen, 500 mg twice per day for 7 days, on the VMB of Rwandan women with BV and/or *T. vaginalis*. We compared their VMB compositions as assessed by microscopy and sequencing pre- and posttreatment, and determined sociodemographic and biological correlates of treatment failure.

## Materials and Methods

Data were collected at the Rinda Ubuzima research clinic in Kigali, Rwanda, in 2015 (flow diagram in Appendix A, Figure A.1). HIV-negative, nonpregnant women, aged 18–45 years, and in good overall physical and mental health, were screened at a pretreatment visit. Recruitment targeted women at high risk of BV/*T. vaginalis*, defined as having had more than 1 sex partner, or having been treated for BV and/or a sexually transmitted infection, in the last 12 months. Women who were BV-positive (by Nugent 7–10 and/or modified Amsel criteria as defined below) and/or *T. vaginalis*-positive (by wet mount and/or by culture), regardless of symptomatology, were treated with 7 days of 500 mg generic oral metronidazole (Tricozole; Laboratory & Allied, Ltd, Nairobi, Kenya) twice daily. Women also were tested, and treated or referred, for pregnancy, HIV, syphilis, *Chlamydia trachomatis*, *Neisseria gonorrhoeae*, vulvovaginal candidiasis, and urinary tract infections, using local guidelines.^20^ Women returned for a posttreatment visit within 3 days after metronidazole treatment completion and were retested for BV, *T. vaginalis*, and vulvovaginal candidiasis. Dacron vaginal swabs for molecular VMB testing were collected during speculum examinations at both visits, and stored dry at –80°C.

### Diagnostic procedures

BV was diagnosed by Gram stain Nugent scoring (a score of 0–3 was considered optimal, 4–6 intermediate microbiota, and 7–10 BV),^8^ and by modified Amsel criteria (defined as the presence of at least 2 of the following criteria: vaginal pH >4.5, positive whiff test, and/or ≥20% clue cells).^9^ Vaginal pH was measured by pressing a pH paper strip (pH range 3.6–6.1 with 0.3 increments; Machery–Nagel, Düren, Germany) against the vaginal wall. *T. vaginalis* was diagnosed when the *T. vaginalis* InPouch culture (Biomed Diagnostics, White City, OR) was positive and/or if motile trichomonads were observed on wet mount. Vulvovaginal candidiasis was diagnosed when budding yeasts and (pseudo)hyphae were seen on wet mount. Other diagnostic testing is described in Appendix A.

### Molecular VMB testing

DNA was extracted from 1 swab per woman per visit (N=136), using lysozyme lysis and bead-beating procedures combined with the Qiagen DNeasy Blood and Tissue kit (Qiagen, Manchester, UK) (Appendix A).^21^ The V3–V4 region of 16S rRNA genes were amplified and sequenced on an Illumina HiSeq 2500 instrument (Illumina, San Diego, CA) run in rapid mode, 2× 300bp using a 250PE and 50PE kit. The panbacterial 16S rRNA gene copy concentration per sample was determined using the BactQuant quantitative PCR assay.^22^

### Molecular data processing

Molecular data processing steps are described in Appendix A. To summarize, DADA2^23^ in R v3.2.3 (R foundation for Statistical Computing 2016, Vienna, Austria) was used to assign reads to amplicon sequence variants (ASVs) using Silva v128 as the reference database,^24^ with additional taxonomic assignments made using other databases (Appendix A). Relative abundances were rarefied at 1111 reads using the *GUniFrac* 1.0 package in R.^25^ The rarefied ASV relative abundance table consisted of 204 ASVs in 134 samples, mapping to species (133; 65.2%), genus (55; 27.0%), or higher taxonomic levels (16; 7.8%). Bacterial cell concentrations in cells/μL per ASV per sample were estimated by multiplying the ASV-specific copy-normalized relative abundance by the sample-specific 16S rRNA gene copies concentration (Appendix A). This yielded estimated concentrations of 204 ASVs in 129 samples, which were log_10_-transformed. Of the 204 ASVs, 108 ASVs were present at a relative abundance of at least 1% in at least 1 sample; the other 96 ASVs were minority species.

Data reduction was required for some biostatistical analyses and was done in 3 different ways. First, Simpson diversity (1-D) was calculated for each sample. Second, each ASV was assigned to 1 of 4 “bacterial groups” based on the published literature (Appendix B): (1) lactobacilli; (2) BV-anaerobes (Actinobacteria, Bacteroidetes, Firmicutes, Fusobacteria, and Tenericutes except those included in the other 3 groups); (3) pathobionts (most Proteobacteria, and streptococci, staphylococci, enterococci, *Spirochaetaceae*, *Listeria*, *C. trachomatis*, and *N. gonorrhoeae*); and (4) “other bacteria” (a rest group, containing Actinobacteria that are known to be [facultative] aerobic skin bacteria, *Bifidobacterium* species, and 7 difficult-to-classify minority species). Within each sample, read counts of ASVs belonging to the same bacterial group were summed (Appendix A). Third, we used hierarchical clustering based on Euclidean distance to pool samples into 7 VMB types: (1) *L. iners*-dominated (Li; >75% lactobacilli of which *L. iners* was the most common; n=45 samples); (2) other lactobacilli-dominated (Lo; also >75% lactobacilli of which *L. jensenii* and *L. gasseri* were the most common; n=2); (3) lactobacilli and anaerobes (LA; 25%–75% lactobacilli; n=30); (4) polybacterial *G. vaginalis*-containing (BV_GV; <25% lactobacilli and 10%–50% *G. vaginalis*; n=30), (5) other polybacterial low-*G. vaginalis* (BV_noGV; <25% lactobacilli and <10% *G. vaginalis*; n=8), (6) *G. vaginalis*-dominated (GV; <25% lactobacilli and >50% *G. vaginalis*; n=12); and (7) pathobionts-containing (PB; >20% pathobionts; n=7). The samples in VMB types 1–6 had a maximum of 0.1%–15.9% pathobionts per VMB type.

### Statistical analyses

Statistical analyses were performed using Stata, version 13 (StataCorp, College Station, TX) and R. VMB characteristics pre- and posttreatment were compared using the Stuart–Maxwell test for matched categorical data, and Wilcoxon signed-rank test for matched continuous data, for all women (N=68), and women stratified by treatment success/failure (women who had Nugent 7–10 pretreatment and a valid Nugent score at posttreatment; N=55), pretreatment *C. trachomatis*/*N. gonorrhoeae* status (results became available after metronidazole treatment completion; N=67 due to 1 missing result), having received another antibiotic in addition to metronidazole at the pretreatment visit or not (N=68), and having reported unusual vaginal discharge pretreatment or not (N=68). Successful treatment was defined as Nugent 7–10 pretreatment and Nugent 0–3 posttreatment, and treatment failure as Nugent 7–10 pretreatment and Nugent 4–10 posttreatment. Kruskal–Wallis test for continuous variables, and Fisher’s exact test for binary variables, were used for cross-sectional comparisons. Bivariable logistic regression was used to investigate associations between individual baseline sociodemographic and biological characteristics and treatment failure.

### Ethical statement

All participants provided written informed consent. The study was conducted in accordance with the Helsinki Declaration, and approved by the National Ethics Committee of Rwanda and the University of Liverpool Research Ethics Subcommittee for Physical Interventions.

## Results

We screened 176 women, and 68 women completed metronidazole treatment (ineligibility reasons in Appendix A, Figure A.1): 82.4% had BV alone, 2.9% had *T. vaginalis* alone, and 14.7% had both BV and *T. vaginalis*. The median age was 31 years (range 19–42), and most were sex workers (Table 1). Thirteen women (26.5%) reported unusual vaginal discharge at the pretreatment visit, but none had proactively sought care for these symptoms before joining the study. Some women (26.5%) received another antibiotic in addition to metronidazole for another condition, or had an ongoing *C. trachomatis* and/or *N. gonorrhoeae* infection during metronidazole treatment (38.2%; Table 1). At the posttreatment visit, all women were BV-negative by modified Amsel criteria and *T. vaginalis*-negative by culture and wet mount, and no women reported urogenital symptoms (including unusual vaginal discharge), adverse events (including vomiting), or social harms. Of the 56 women with Nugent 7–10 pretreatment, 30 (54.5%) had Nugent 0–3, 11 (20.0%) Nugent 4–6, and 14 Nugent 7–10 posttreatment (Table 2).

**Table 1:** Participant characteristics

Characteristics	Pretreatment (N=68)	Posttreatment (N=68)
Sociodemographics and sexual behavior		
Age, y, median [IQR]	31 [27–35]	NA
Marital status, n (%)		NA
Never married	50 (73.5)	
Married	5 (7.4)	
Divorced	12 (17.6)	
Widowed	1 (1.5)	
Education level, n (%)		NA
No schooling	14 (20.6)	
Primary school not completed	31 (45.6)	
Primary school completed	17 (25.0)	
Secondary school not completed	6 (8.8)	
Secondary school completed	0	
Number of sex partners in lifetime, n, median [IQR]	30 [7–463]	NA
Number of sex partners in last 12 months (pretreatment) or month (posttreatment), n, median [IQR]	11 [4–152]	5 [3–15.5]^a^
Exchanged sex for money/goods in past month, n (%)	63 (92.6)	60 (92.3)^a^
Vaginal sex frequency last 2 weeks, n, median [IQR]	12 [8–18]	11 [8–19]^a^
Any condom use in past 2 weeks, n (%)		
Always	14 (20.6)	23 (33.8)^a^
Sometimes but not always	51 (75.0)	36 (52.9)
Never	3 (4.4)	6 (8.8)
No sex in the past 2 weeks	0	3 (4.4)
Condom use during last sex act, n (%)	36 (52.9)	44 (64.7)^a^
Currently using hormonal contraception, n (%)	42 (61.8)	42 (62.7)^a^
Currently breastfeeding, n (%)	14 (21.2)^a^	NA
Inserted anything inside the vagina in the last 12 months, n (%)	NA	26 (38.2)
Had menses in the 7 days prior to the visit, n (%)	NA	11 (16.2)
Any current urogenital symptoms (at pretreatment visit, including last 2 weeks), patient-reported, n (%)	49 (72.1)	0
Current unusual vaginal discharge (at pretreatment visit, including last 2 weeks), patient-reported, n (%)	13 (26.5)	0
Received antibiotic in addition to metronidazole at pretreatment visit, n (%)^b^	18 (26.5)	NA
Received antifungal treatment at pretreatment visit, n (%)^c^	6 (8.8)	NA
Laboratory results		
HIV by serology, n (%)^d^	0	NA
Positive urine pregnancy test, n (%)^d^	0	0^a^
BV by Nugent 7–10, n (%)	56 (83.6)^a^	17 (25.8)
BV by modified Amsel criteria,^e^ n (%)	49 (72.1)	0
*Trichomonas vaginalis* on wet mount, n (%)	6 (8.8)^a^	0
*T. vaginalis* by InPouch culture, n (%)	11 (16.4)	0
Yeasts on wet mount, n (%)	6 (8.9)	4 (5.9)
Positive urinalysis test, n (%)	17 (25.0)	0^a^
Syphilis by serology, n (%)	4 (5.9)	NA
Positive herpes simplex virus type 2 serology, n (%)	44 (64.7)	NA
*Chlamydia trachomatis* by PCR, n (%)	20 (29.4)	NA
*Neisseria gonorrhoeae* by PCR, n (%)	13 (19.1)	NA

*BV*, bacterial vaginosis; *IQR*, interquartile range; *NA*, not assessed; *PCR*, polymerase chain reaction.

*Verwijs et al. Impact of oral metronidazole treatment on the vaginal microbiota and correlates of treatment failure. Am J Obstet Gynecol 2020*.

a 1–3 missing values

b Includes ciprofloxacin for urinary tract infection and penicillin for syphilis

c Three women received both an antifungal and another antibiotic in addition to metronidazole

d All enrolled participants were HIV-negative and nonpregnant by design

e Two or more positive of vaginal pH >4.5, positive whiff test, and/or ≥20% clue cells observed on wet mount.

**Table 2:** VMB characteristics before and after metronidazole treatment, including stratification by treatment success/failure

VMB outcomes	All participants			Successful treatment^b^			Treatment failure^b^		
	Pretreatment (N=68)	Posttreatment (N=68)	*P* value^a^	Pretreatment (n=30)	Posttreatment (n=30)	*P* value^a^	Pretreatment (n=25)	Posttreatment (n=25)	*P* value^a^
Nugent categories (n %)^c^			<.001			NA^d^			NA^d^
0–3	5 (7.5)	36 (54.6)		0	30 (100)		0	0	
4–6	6 (9.0)	13 (19.7)		0	0		0	11 (44.0)	
7–10	56 (83.6)	17 (25.8)		30 (100)	0		25 (100)	14 (56.0)	
Mean inverse Simpson diversity index (95% CI)^e^	0.67 (0.60–0.73)	0.31 (0.25–0.38)	<.001	0.70 (0.61–0.80)	0.13 (0.06–0.21)	<.001	0.77 (0.70–0.85)	0.47 (0.39–0.56)	.001
VMB type, n (%)^e^			<.001			<.001			.002
Li	10 (14.9)	35 (52.2)		2 (6.9)	23 (79.3)		1 (4.0)	6 (24.0)	
Lo	0	2 (3.0)		0	1 (3.5)		0	0	
LA	12 (17.9)	18 (26.9)		7 (24.1)	2 (6.9)		2 (8.0)	14 (56.0)	
BV_GV	28 (41.8)	2 (3.0)		13 (44.8)	0		14 (56.0)	2 (8.0)	
BV_noGV	8 (11.9)	0		4 (13.8)	0		3 (12.0)	0	
GV	8 (11.9)	4 (6.0)		3 (10.3)	1 (3.5)		5 (20.0)	2 (8.0)	
PB	1 (1.5)	6 (9.0)		0	2 (6.9)		0	1 (4.0)	
Vaginal pH, median [IQR]	5.3 [5.0–5.6]	4.4 [3.6–4.6]	<.001	5.3 [5.0–5.6]	4.1 [3.6–4.4]	<.001	5.6 [5.0–5.6]	4.4 [4.4–4.7]	<.001
Vulvovaginal candidiasis, n (%)	6 (8.8)	4 (5.9)	.527	1 (3.3)	3 (10.0)	.317	1 (4.0)	1 (4.0)	1.00
Bacterial group relative abundances, mean (95% CI)^e^									
Total lactobacilli	0.24 (0.15–0.32)	0.72 (0.64–0.80)	<.001	0.18 (0.08–0.27)	0.88 (0.78–0.98)	<.001	0.10 (0.02–0.18)	0.56 (0.45–0.68)	<.001
Total BV-anaerobes	0.75 (0.67–0.83)	0.23 (0.16–0.30)	<.001	0.81 (0.71–0.91)	0.07 (0–0.15)	<.001	0.89 (0.81–0.97)	0.40 (0.29–0.52)	<.001
Total pathobionts	0.02 (0.01–0.03)	0.05 (0.02–0.09)	.050	0.01 (0–0.02)	0.05 (–0.02 to 0.11)	.118	0.01 (0–0.03)	0.03 (0.01–0.05)	.173
Total other bacteria	0 (0–0)	0 (0–0)	.674	0 (0–0)	0 (0–0)	.173	0 (0–0)	0 (0–0)	.764
Bacterial group concentrations in log_10_ cells/μL, mean (95% CI)^f^									
Total bacteria	6.59 (6.39–6.78)	5.85 (5.66–6.04)	<.001	6.59 (6.31–6.86)	5.65 (5.38–5.91)	<.001	6.68 (6.36–7.01)	6.23 (5.93–6.54)	.028
Total lactobacilli	4.98 (4.61–5.35)	5.56 (5.34–5.78)	.017	4.92 (4.36–5.49)	5.47 (5.16–5.77)	.124	4.62 (3.92–5.31)	5.80 (5.38–6.21)	.001
Total BV-anaerobes	6.23 (5.88–6.57)	4.55 (4.14–4.95)	<.001	6.46 (6.14–6.78)	3.81 (3.23–4.38)	<.001	6.62 (6.26–6.97)	5.79 (5.45–6.13)	.003
Total pathobionts	1.92 (1.36–2.48)	2.01 (1.48–2.54)	.939	1.09 (0.32–1.87)	1.48 (0.74–2.21)	.649	2.30 (1.40–3.19)	2.66 (1.65–3.66)	.637
Total other bacteria	1.85 (1.36–2.35)	1.46 (1.01–1.92)	.176	1.71 (0.96–2.45)	0.91 (0.36–1.47)	.043	2.44 (1.56–3.31)	2.34 (1.41–3.27)	.525
Individual bacteria concentrations in log_10_ cells/μL, mean (95% CI)^f^									
*L. iners*	4.81 (4.38–5.24)	5.28 (4.94–5.62)	.072	4.91 (4.34–5.48)	5.10 (4.54–5.65)	.501	4.27 (3.89–5.14)	5.63 (5.10–6.17)	<.001
*L. crispatus*^g^	0.15 (–0.02 to 0.33)	0.51 (0.16–0.85)	.089	0 (0–0)	0.47 (–0.08 to 1.01)	.083	0.25 (–0.12 to 0.62)	0.55 (–0.08 to 1.19)	.330
Other lactobacilli^h^	1.46 (0.97–1.94)	3.03 (2.57–3.48)	<.001	0.67 (0.15–1.19)	2.62 (1.93–3.31)	<.001	1.18 (0.45–1.91)	3.31 (2.45–4.17)	.001
*Gardnerella vaginalis*	5.62 (5.20–6.03)	4.12 (3.63–4.61)	<.001	6.00 (5.69–6.31)	3.29 (2.62–3.96)	<.001	6.11 (5.74–6.47)	5.66 (5.30–6.01)	.115
*Atopobium vaginae*	4.58 (4.00–5.16)	1.54 (1.06–2.02)	<.001	4.91 (4.14–5.67)	1.44 (0.76–2.11)	<.001	5.43 (4.83–6.03)	1.76 (0.78–2.73)	<.001
*Prevotella* species	4.67 (4.18–5.16)	1.35 (0.90–1.79)	<.001	5.07 (4.59–5.55)	1.31 (0.71–1.90)	<.001	5.24 (4.47–6.00)	1.62 (0.69–2.54)	<.001
*Sneathia* species	4.18 (3.63–4.73)	1.08 (0.63–1.54)	<.001	4.38 (3.64–5.14)	1.10 (0.45–1.76)	<.001	4.90 (4.16–5.63)	1.43 (0.51–2.36)	<.001
*Megasphaera* species	3.17 (2.56–3.79)	0.22 (-0.01–0.44)	<.001	3.96 (3.10–4.81)	0 (0–0)	<.001	3.34 (2.32–4.35)	0.55 (–0.09 to 1.18)	.001
*Veillonella* species	2.37 (1.75–3.00)	0.28 (0.01–0.56)	<.001	1.85 (0.89–2.81)	0.27 (–0.12 to 0.66)	.005	2.83 (1.79–3.87)	0.22 (–0.24 to 0.68)	.002
BVAB1	1.76 (1.11–2.42)	0.46 (0.15–0.77)	<.001	2.08 (0.97–3.19)	0.34 (0.01–0.67)	.002	2.00 (0.84–3.15)	0.75 (–0.02 to 1.53)	.067
*Fusobacterium* species	0.53 (0.17–0.89)	0 (0–0)	.008	0.44 (–0.06 to 0.95)	0 (0–0)	.008	0.68 (0.02–1.34)	0 (0–0)	.046
*Streptococcus* species	1.47 (0.92–2.02)	1.34 (0.84–1.85)	.453	0.83 (0.10–1.55)	0.85 (0.18–1.52)	.286	1.76 (0.83–2.68)	2.05 (1.06–3.05)	.767
*Staphylococcus* species	0.26 (0.05–0.47)	0.60 (0.27–0.93)	.655	0.09 (–0.10 to 0.29)	0.34 (–0.01 to 0.70)	ND	0.34 (–0.05 to 0.72)	0.87 (0.12–1.63)	.317
*Escherichia/Shigella* species	0.10 (–0.04 to 0.25)	0.86 (0.45–1.27)	.317	0.24 (–0.10 to 0.59)	0.41 (0.00–0.83)	.317	0 (0–0)	1.41 (0.52–2.29)	ND

*BV*, bacterial vaginosis; *BVAB1*, BV-associated bacterium type 1; *BV_GV*, polybacterial *Gardnerella vaginalis*-containing; *BV_noGV*, polybacterial but low *G. vaginalis*; *CI*, confidence interval; *GV*, *G. vaginalis*-dominated; *LA*, lactobacilli and anaerobes; *Li*, *L. iners*-dominated; *Lo*, other lactobacilli-dominated; *NA*, not applicable; *ND*, not determinable; *PB*, pathobionts-containing; *VMB*, vaginal microbiota.

*Verwijs et al. Impact of oral metronidazole treatment on the vaginal microbiota and correlates of treatment failure. Am J Obstet Gynecol 2020*.

a Stuart–Maxwell test for matched categorical data and Wilcoxon signed-rank test for matched continuous data

b Successful treatment was defined as having a Nugent score of 7–10 before treatment and 0–3 after treatment (n=30), whereas treatment failure was defined as having a Nugent score of 7–10 before treatment and 4–10 after treatment (n=25). Thirteen women were excluded from these analyses because they did not have Nugent 7–10 at the pretreatment visit (n=12) or did not have a valid Nugent result at the posttreatment visit (n=1)

c Valid Nugent data available for 67 participants at the pretreatment visit and 66 participants at the posttreatment visit

d The definition of treatment success/failure was based on Nugent scores and these *P* values are therefore meaningless

e Relative abundance, Simpson diversity indices, and VMB type data available for 67 participants at each visit

f Concentration data may contain at most 5 missing values (see Appendix A Methods)

g Includes all amplicon sequence variants attributed to *L. crispatus*, also those with multiple species assignments

h Includes amplicon sequence variants attributed to *L. jensenii*, *L. delbrueckii*, *L. fermentum*, *L. gasseri*, *L. johnsonii*, and *Lactobacillus genus*, as well as 11 other minority amplicon sequence variants.

**Figure 1:**
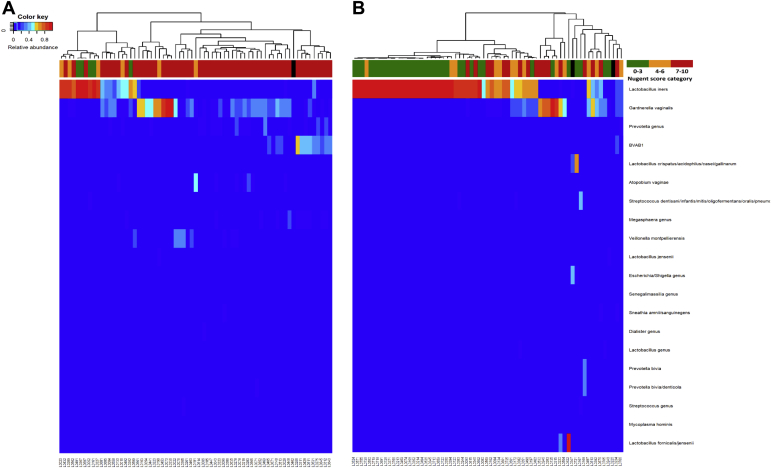
Heatmaps of 16S rRNA gene sequencing data pre- and posttreatment **A–B,** Heatmaps at the pretreatment (**A**) and the posttreatment visit (**B**) depicting the 20 amplicon sequence variants with the greatest mean relative abundance on the y-axis and samples (N=67 at each visit) on the x-axis. The *dendrogram* above the heatmap depicts vaginal microbiota clusters based on Euclidean distance. The *bar* below the dendrogram depicts Nugent score categories (see legend; *black* means no score available). *BVAB1*, bacterial vaginosis-associated bacterium type 1. *Verwijs et al. Impact of oral metronidazole treatment on the vaginal microbiota and correlates of treatment failure. Am J Obstet Gynecol 2020.*

**Figure 2:**
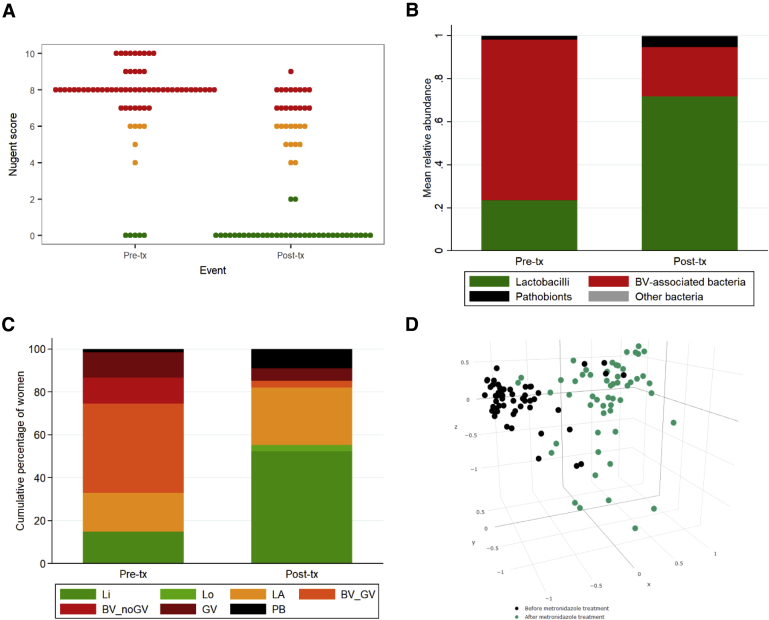
Nugent scores, mean bacterial group relative abundances, and vaginal microbiota types pre- and posttreatment **A–C,** Changes in vaginal microbiota characteristics before and after metronidazole treatment. **A,** Nugent scores, **B,** mean bacterial group relative abundances, and **C,** vaginal microbiota types. **D,** Three-dimensional non-metric multidimensional scaling plot based on rarefied relative abundances of samples before and after metronidazole treatment. The figure shows that samples cluster together by visit (and hence, treatment status). *BV*, bacterial vaginosis; *BV_GV*, polybacterial *Gardnerella vaginalis*-containing; *BV_noGV*, polybacterial but low *G. vaginalis*; *GV*, *G. vaginalis*-dominated; *IQR*, interquartile range; *LA*, lactobacilli and anaerobes; *Li*, *Lactobacillus iners*-dominated; *Lo*, other lactobacilli-dominated; *PB*, pathobionts-containing; *Post-tx*, posttreatment visit; *Pre-tx*, pretreatment visit. *Verwijs et al. Impact of oral metronidazole treatment on the vaginal microbiota and correlates of treatment failure. Am J Obstet Gynecol 2020.*

Pre- and posttreatment 16S microbiota data (Figure 1) shows a shift toward increased relative abundance of lactobacilli (mainly *L. iners*) and decreased relative abundances of several BV-anaerobes (Figure 1). The mean bacterial group relative abundance data confirmed this (Table 2, Figure 2, B) and additionally showed that the mean relative abundance of pathobionts increased posttreatment (Wilcoxon signed rank *P*=.050). Metronidazole treatment was associated with a significant decrease in the mean concentration of total bacteria from 6.59 log_10_/μL pretreatment to 5.85 log_10_/μL posttreatment (*P*<.001; Table 2). The mean BV-anaerobes concentration decreased from 6.23 log_10_/μL to 4.55 log_10_/μL (*P*<.001), the mean *Lactobacillus* concentration increased from 4.98 log_10_/μL to 5.56 log_10_/μL (*P*=.017), and the mean concentrations of pathobionts (1.92 log_10_/μL pretreatment and 2.01 log_10_/μL posttreatment; *P*=.939) and “other bacteria” (1.85 log_10_/μL pretreatment and 1.46 log_10_/μL posttreatment; *P*=.176) did not change significantly. Among lactobacilli, the concentrations of *L. iners*, *L. crispatus*, and “other lactobacilli” (mostly *L. jensenii* and *L. gasseri*) all increased, with *L. iners* having the greatest concentrations before and after treatment, but “other lactobacilli” achieving the greatest concentration increase (Table 2). The median vaginal pH decreased from 5.3 to 4.4 (*P*<.001). Among BV-anaerobes, the concentrations of 8 common BV-anaerobes that were individually tested (*Gardnerella*, *Atopobium*, *Prevotella*, *Sneathia*, *Megasphaera*, *Veillonella*, and *Fusobacterium* species, and BV-associated bacterium type 1) decreased (Table 2). The concentrations of the 3 most common pathobionts in our dataset (*Streptococcus*, *Staphylococcus*, *Escherichia/Shigella* species) did not change significantly (Table 2).

**Figure 3:**
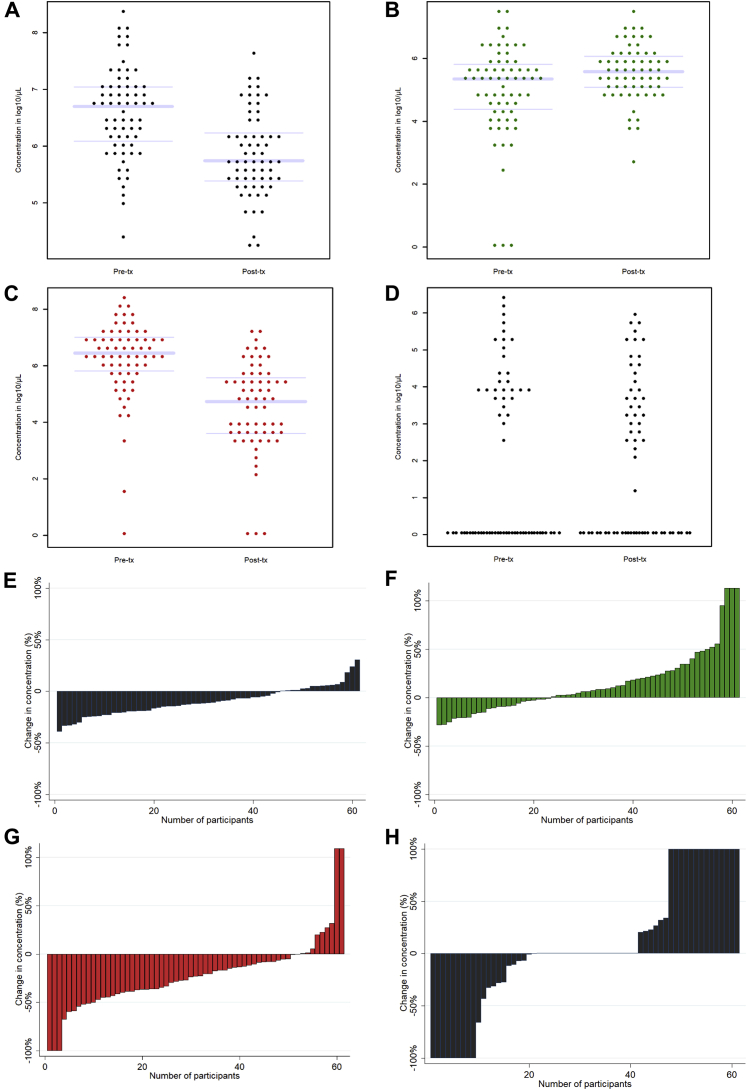
Individual bacterial group concentrations pre- and posttreatment **A-D,** Changes in total bacterial concentrations and bacterial group concentrations before (n=66) and after metronidazole treatment (n=63). **A,** Total bacterial concentration, **B,** total lactobacilli, **C,** total BV-anaerobes, and **D,** total pathobionts (boxplot not shown because of high proportion of zero values). See Table 2 for mean concentrations and 95% confidence intervals, and statistical significance. **E-H,** Change in concentrations between pre- and posttreatment, expressed as a percentage for every individual participant with valid quantitative polymerase chain reaction results at both visits (n=61), for total bacterial concentration (**E**), total *Lactobacillus* (**F**), total BV-anaerobes (**G**), and total pathobionts (**H**). In some women, concentration went from zero to non-zero; these increases were set at 100% or the greatest increase observed among the other participants, whichever was greatest. *BV*, bacterial vaginosis; *conc*, concentration; *Post-tx*, posttreatment visit; *Pre-tx*, pretreatment visit. *Verwijs et al. Impact of oral metronidazole treatment on the vaginal microbiota and correlates of treatment failure. Am J Obstet Gynecol 2020.*

Although the mean trends were clear, the interindividual variability was high (Figure 3, A–G). Of the 61 participants for whom pre- and posttreatment concentration data were available, most had decreases in total bacterial concentration (n=45) and BV-anaerobes (n=52), but not everyone (Figure 3, E and G). BV-anaerobes were completely eradicated in only 3 women (4.9%), and the concentration was reduced by more than 50% in an additional 7 women (11.5%). Pathobiont concentrations showed the most interindividual variability (Figure 3, H).

The mean inverse Simpson diversity index was 0.67 pretreatment and 0.31 posttreatment (*P*<.001; Table 2). Metronidazole treatment changed the proportions of women with certain VMB types based on hierarchical clustering results: the proportions of women with lactobacilli-dominated VMB types (Li and Lo) and the mixed LA VMB type increased, whereas the proportions of women with the 3 BV-associated VMB types (BV_GV, BV_noGV, and to a lesser extent GV) decreased (Table 2, Figure 2, C). The number of women with a pathobionts-containing VMB type increased from 1 (1.5%) pretreatment to 6 (9.0%) posttreatment. Alluvial diagrams show that the majority of women transitioned from the 3 BV-associated VMB types into VMB types containing lactobacilli (Li, Lo, and LA; Figures A.2, A and B). However, women with a BV_GV or BV_noGV VMB type transitioned nonsignificantly more often into a lactobacilli-dominated VMB type (16/27 [59.3%], and 4/8 [50.0%], respectively) than women with a GV VMB type (1/8 [12.5%]; Fisher’s exact *P*=.084 comparing the 3 groups; Figure A.2, A).

Participants with treatment failure as defined by Nugent scoring had a lower mean lactobacilli relative abundance, smaller decreases in mean total bacteria and BV-anaerobes concentrations, and less often a lactobacilli-dominated VMB type, posttreatment (Appendix A, Figure A.3, A–D). They also had a greater mean pathobionts concentration pretreatment (Appendix A, Table A.1). Successfully treated participants had significant decreases in the mean concentrations of all 8 common BV-anaerobes in our dataset that were tested individually, but unsuccessfully treated participants did not have decreases in *G. vaginalis* and BVAB1 (Table 2). Simpson diversity did not differ by treatment success/failure (Appendix A: Figure A.3, E). In logistic regression models, we did not identify any statistically significant sociodemographic or biological correlates of treatment failure, except for pathobionts concentration pretreatment (*P*=.044; Table 3). Pretreatment *C. trachomatis*/*N. gonorrhoeae* status, having received another antibiotic in addition to metronidazole, and reporting unusual vaginal discharge at the pretreatment visit, did not modify the effects of metronidazole treatment on the VMB (Appendix A: Tables A.2, A.3, A.4; Figures A.4, A.5, A.6).

**Table 3:** Sociodemographic and biological correlates of metronidazole treatment success^a^

	OR (95% CI)^b^	*P* value^b^
Sociodemographic correlates		
Age (continuous variable)	1.06 (0.97–1.17)	.186
Education level		
Primary school not completed^c^	1.23 (0.26–5.90)	.795
Primary school completed^c^	1.80 (0.31–10.5)	.514
Secondary school not completed^c^	0.33 (0.02–4.74)	.417
Exchanged sex for money/goods in month prior to posttreatment visit	5.40 (0.56–52.08)	.145
Used a condom at the last vaginal sex act prior to posttreatment visit	0.99 (0.97–1.02)	.678
Condom use in the 2 weeks prior to posttreatment visit		
Always vs never	0.36 (0.03–3.92)	.399
Sometimes (but not always) vs never	0.22 (0.02–2.19)	.196
Currently using hormonal contraception at the posttreatment visit	0.69 (0.23–2.05)	.499
Currently breastfeeding at the posttreatment visit	1.00 (0.97–1.03)	.874
Inserted anything in vagina in the 12 months prior to posttreatment visit	1.03 (0.34–3.10)	.959
Had menses in 7 days prior to posttreatment visit	1.83 (0.41–8.23)	.429
Reported any urogenital symptoms at the pretreatment visit	0.67 (0.21–2.11)	.496
Reported any urogenital symptoms at the posttreatment visit	NA^d^	NA^d^
Biological correlates (at pretreatment visit)		
Total lactobacilli concentration	1.14 (0.80–1.62)	.476
Total BV-anaerobes concentration	0.79 (0.41–1.53)	.489
Total pathobionts concentration	0.76 (0.58–0.99)	.044
Total other bacteria concentration	0.83 (0.63–1.10)	.191
*Gardnerella vaginalis* concentration^e^	0.86 (0.44–1.65)	.640
*Atopobium vaginae* concentration^f^	0.83 (0.59–1.17)	.285
*Prevotella* concentration	0.93 (0.65–1.33)	.691
*Sneathia* concentration	0.86 (0.63–1.16)	.318
*Megasphaera* concentration	1.12 (0.89–1.42)	.333
*Veillonella* concentration	0.85 (0.68–1.06)	.157
BVAB1 concentration	1.01 (0.83–1.23)	.912
*Fusobacterium* concentration	0.89 (0.61–1.30)	.549
Vaginal microbiota type		
LA vs Li	1.75 (0.10–30.84)	.702
BV_GV vs Li	0.46 (0.04–5.75)	.550
BV_noGV vs Li	0.67 (0.04–11.29)	.779
GV vs Li	0.30 (0.02–4.91)	.398
BV_GV vs LA	0.27 (0.05–1.52)	.136
BV_noGV vs LA	0.38 (0.04–3.34)	.383
GV vs LA	0.17 (0.02–1.44)	.104
Pooled vaginal microbiota type		
LA vs *Lactobacillus*-dominated [Li or Lo]	1.75 (0.10–30.84)	0.702
[BV_GV or BV_noGV or GV] vs *Lactobacillus*-dominated [Li or Lo]	0.45 (0.04–5.40)	0.532
Yeasts on wet mount	0.83 (0.05–13.9)	0.896
*Trichomonas vaginalis* on wet mount	1.71 (0.15–20.1)	0.661
Positive urinalysis test	1.22 (0.33–4.44)	0.765
Any bacterial sexually transmitted infection (CT/NG/syphilis)	0.65 (0.22–1.91)	0.435
Positive herpes simplex virus type 2 serology	2.16 (0.70–6.69)	0.178

*BV*, bacterial vaginosis; *BVAB1*, BV-associated bacterium type 1; *BV_GV*, polybacterial *Gardnerella vaginalis*-containing; *BV_noGV*, polybacterial but low *G. vaginalis*; *CI*, confidence interval; *CT*, *Chlamydia trachomatis*; *GV*, *G. vaginalis*-dominated; *LA*, lactobacilli and anaerobes; *Li*, *L. iners*-dominated; *Lo*, other lactobacilli-dominated; *NA*, not applicable; *NG*, *Neisseria gonorrhoeae*; *OR*, odds ratio.

*Verwijs et al. Impact of oral metronidazole treatment on the vaginal microbiota and correlates of treatment failure. Am J Obstet Gynecol 2020*.

a Successful treatment defined as Nugent 7–10 pre- and 0–3 posttreatment (n=30 women) and treatment failure as Nugent 7–10 pre and 4–10 posttreatment (n=25 women)

b Bivariable logistic regression models

c Compared with no schooling

d No women reported urogenital symptoms at the posttreatment visit

e Includes reads assigned to *Gardnerella* genus

f Includes reads assigned to *Atopobium* genus.

## Discussion

### Principal findings

In this study among high-risk women in Rwanda diagnosed with BV and/or *T. vaginalis*, the cure rate of 7-day oral metronidazole treatment by Nugent scoring was only 54.5%. The sequencing data showed a decrease in BV-anaerobes (but a reduction of more than 50% in only 16.4% of women), an increase in lactobacilli, and no change in pathobionts. Treatment failure was associated with greater levels of pretreatment *Gardnerella vaginalis* or pathobionts levels but not with sociodemographic factors.

### Results of the study in context of what is known

Metronidazole treatment resulted in a mean BV-anaerobes concentration reduction (as well as *T. vaginalis* eradication), which is in agreement with a priori knowledge about the mechanism of action of metronidazole.^15^, ^16^ However, the extent of BV-anaerobes reduction was more modest than expected, with only 16.4% of women having a reduction of more than 50%. The mean lactobacilli concentration increased, and mean concentrations of pathobionts and “other bacteria” were low pre- and posttreatment, resulting in an overall 5.5-fold reduction of total bacterial concentration (from 6.59 to 5.85 log_10_/μL). The observed increase in lactobacilli is consistent with culture studies showing that lactobacilli are not sensitive to metronidazole^17^ but is inconsistent with claims made by some clinical researchers that the high BV recurrence rate may be due to detrimental effects of metronidazole on lactobacilli.^14^ The reduction in BV-anaerobes concentration clearly allows lactobacilli, which are not affected by metronidazole, to expand.^26^, ^27^, ^28^ In our study population of high-risk Rwandan women, *L. iners* was by far the most common *Lactobacillus* species pre- and posttreatment (4.81 and 5.28 log_10_/μL, respectively), “other lactobacilli” (which includes *L. jensenii*) increased the most during treatment (from 1.46 to 3.03 log_10_/ μL), and *L. crispatus* was uncommon and increased only slightly (from 0.15 to 0.51 log_10_/μL). A metronidazole study in American women also showed that *L. iners* and *L. jensenii* concentrations increased more than the *L. crispatus* concentration.^29^

Women with a pretreatment VMB type containing a relative abundance of >50% *G. vaginalis*, compared with ≤50%, were more likely to continue to have a dysbiotic VMB type posttreatment, but pretreatment *G. vaginalis* concentration (as a continuous variable) was not associated with achieving Nugent 0–3 posttreatment. Both findings are consistent with earlier studies,^13^, ^29^ and with the *G. vaginalis*-containing biofilm hypothesis of treatment failure.^10^ Metronidazole may eliminate dispersed *G. vaginalis* at low to modest concentrations but may no longer be able to do so when a biofilm (containing a high concentration of *G. vaginalis*) has been established. However, other hypotheses have also been posited. A recent metatranscriptomics study showed that the VMB of BV patients with treatment failure contained *G. vaginalis* with upregulated clustered regularly interspaced short palindromic repeat-associated (CRISPR)-genes, which may protect the bacteria against metronidazole.^30^ In our study, the concentrations of all other key BV-associated bacteria, including *A. vaginae*, were effectively reduced by metronidazole and were not associated with treatment failure. This is in accordance to one study,^29^ but in contrast to others that have suggested that pretreatment presence or concentration of *A. vaginae* was associated with treatment failure.^31^, ^32^, ^33^

The concentrations of the pathobionts bacterial group were low pre- and posttreatment (1.92 and 2.01 log_10_/μL, respectively). However, pathobionts have greater pathogenicity potential than BV-anaerobes^3^ and may therefore be clinically relevant even at low concentrations. Unfortunately, it is currently unknown which concentrations of which pathobionts in the vagina should be treated to prevent complications, such as transmission to neonates. In our study, metronidazole treatment did not change the pathobionts concentration but did increase the relative abundance due to the reduction in total bacterial concentration. Furthermore, a greater pretreatment pathobionts concentration was associated with increased likelihood of treatment failure. None of the sociodemographic factors that have been associated with VMB composition in other studies, including menses in the 7 days prior to the posttreatment visit,^34^ were associated with treatment failure in our study.

### Research and clinical implications

Our study has several implications. Women with persistent or recurrent BV might benefit from vaginal biofilm-disrupting treatment, adjuvant therapy with lactobacilli-based live biotherapeutics, or treatment with drugs that specifically target all *G. vaginalis* clades. The former 2 are actively researched,^10^, ^35^, ^36^ but the latter are not yet available. Whether these strategies are efficacious in real life would have to be evaluated in clinical trials. Furthermore, diagnostic tests to determine the presence of a biofilm or concentrations of *G. vaginalis* are not yet available to clinicians. Women at risk of complications caused by vaginal pathobionts (not just Group B streptococci), such as pregnant women, might benefit from targeted screening and treatment. We encourage the incorporation of quantitative molecular characterization of both key individual bacteria with pathogenic potential, as well as bacterial communities and biofilms, in future intervention studies.

### Strengths and limitations of the study

Limitations of our study include potentially limited generalizability of the results to lower risk and non-African populations,^37^ and the lack of vaginal biofilm detection and characterization pre- and posttreatment. Recent studies have shown good correlations between the method that we used to quantify relative abundance data and species-specific quantitative polymerase chain reaction results of non-minority species,^38^, ^39^ but additional validation studies are desirable. Although Nugent-based studies have shown that oral and vaginal metronidazole of similar dose and duration of use have similar efficacy for BV,^11^, ^12^ molecular studies comparing the in-depth microbiological effects of different metronidazole formulations are desirable. A major strength of our study is that we used multiple laboratory and analytic methods to characterize the VMB, including methods that incorporated a priori knowledge about the pathogenic potential of specific microorganisms and the types of communities in which they typically live.

## Conclusion

Oral metronidazole treatment alone may not be sufficient for women with recurrent BV or for women at risk of complications caused by pathobionts (such as pregnant women). Additional treatments are urgently needed, including biofilm-disrupting treatments and drugs that specifically target all *G. vaginalis* clades or pathobionts.
