# Supplementary material for: Impact of oral metronidazole treatment on the vaginal microbiota and correlates of treatment failure
Source: Am J Obstet Gynecol. 2020 Feb;222(2):157.e1–157.e13. doi: 10.1016/j.ajog.2019.08.008 (PMC6995998; doi:10.1016/j.ajog.2019.08.008)
Supplement: Appendix A [file mmc1.docx]

**Appendix A**

**Impact of Oral Metronidazole Treatment on the Vaginal Microbiota**

**and Correlates of Treatment Failure**

Marijn C. VERWIJS (MD); Stephen K. AGABA (MD); Alistair C. DARBY (MSc PhD); Janneke H.H.M VAN DE WIJGERT (MD PhD MPH)

**Supplementary Methods**

We conducted a randomized pilot clinical trial to determine safety, preliminary efficacy, acceptability, and feasibility of one antibiotic and two vaginal probiotic interventions to prevent BV recurrence in women diagnosed and treated for bacterial vaginosis (BV) and/or *Trichomonas vaginalis* (TV) in Kigali, Rwanda. In this manuscript, we report the vaginal microbiota (VMB) effects of the initial metronidazole treatment for BV/TV prior to randomization. The other clinical trial results will be published elsewhere. A flow diagram of the entire trial is shown in Figure A.1. We chose to treat women with BV and/or TV with seven days of 500 mg generic oral metronidazole (Tricozole; Laboratory & Allied ltd, Nairobi, Kenya) twice daily. Previous studies suggest that oral and vaginal metronidazole of similar dose and duration of use have similar efficacy for BV,^1,2^ but none of the vaginal metronidazole gels that were available on the market when we designed our study had proven stability at 30°C. We chose to include both women with BV and/or TV as the treatment to these two conditions is the same, and BV and TV are closely interlinked.^3^

*Additional diagnostic testing*

All diagnostic testing was conducted onsite at the Rinda Ubuzima clinic or at the National Reference Laboratory in Kigali. Diagnostic testing for BV, TV, and vulvovaginal candidiasis (VVC) is described in the main manuscript. Whole blood was tested for HIV 1/2 using the Kehua HIV Rapid Test (Kehua Bio-engineering, Shanghai, China), followed by the Alere Determine HIV-1/2 Rapid Test (Abbott Laboratories, Tokyo, Japan) for confirmation of positive results and the Unigold HIV Rapid Test (Trinity Biotech, Bray, Ireland) as tie-breaker (if applicable). Urine was tested for pregnancy using an hCG, and for urinary tract infection using a urinalysis, dipstick test (both by Nova, Atlast Link Technology, Beijing, China). Endocervical swabs were tested for *Chlamydia trachomatis* (CT)/Neisseria *gonorrhoeae* (NG) by real-time Polymerase Chain Reaction (PCR; Presto, Beek, The Netherlands).^4^ Plasma was tested for herpes simplex virus type 2 serology (Kalon, Guildford, UK; using an optical density cut-off of >1.1 for a positive result and <0.9 for a negative result) and syphilis by Rapid Plasma Reagin test followed by *T. pallidum* Hemagglutination Assay (both by Spinreact, Girona, Spain).

*Rationale for 16S rRNA gene sequencing of the VMB before and after metronidazole treatment*

It has long been recognized that BV is a polybacterial condition. However, it has been difficult to define its precise microbiological nature due to the limitations of microscopy and culture.^3,5^16S rRNA sequencing has revolutionized the field. The 16S gene is unique to bacteria. It contains highly preserved regions that can be used to quantify and amplify all 16S genes present in a vaginal sample, as well as variable regions that can be sequenced and then used to determine which bacteria are present.^6^ Many sequencing studies have been conducted since the turn of the century, and these revealed that VMBs are usually dominated by five main *Lactobacillus* species (of which *L. crispatus* and *L. iners* are the most common).^5^ The most common type of vaginal dysbiosis is BV, and the long list of anaerobes that are typically associated with BV have now been well characterized (we refer to them as BV-anaerobes in our manuscript and list them in Appendix B).^5^ Occasionally, bacteria other than lactobacilli or BV-anaerobes are identified, including pathobionts, skin bacteria, and Bifidobacteria.^5^ These bacteria have not yet been very well characterized epidemiologically and clinically. Pathobionts are bacteria that have a higher pathogenicity than BV-anaerobes, and are often associated with hospital and neonatal infections, including Proteobacteria, streptococci, staphylococci, and enterococci.^7^ We assessed pathobionts separately from BV-anaerobes, because they seem to behave differently (although more research is needed to evaluate this properly) and are not typically treated with metronidazole when they cause hospital or neonatal infections.

*DNA extraction*

Dacron vaginal swabs taken by the physician at the pre- and post-treatment visits were frozen dry at -80 °C. Frozen samples were shipped to Liverpool on dry ice. DNA extraction and sequencing were done at the University of Liverpool Centre for Genomic research.^8^ DNA was extracted from one sample per participant per time point (N=136 swabs). The samples were thawed, and DNA was extracted from each sample by adding 180 μl of enzymatic lysis buffer containing lysozyme (Sigma-Aldrich, Dorset, UK); incubation for 30 minutes at 37 ^0^C; adding 25 μl of proteinase K and 200 μl of buffer AL using the Qiagen DNeasy Blood and Tissue kit (Qiagen, Manchester, UK); incubation for 30 minutes at 56^0^ C; and bead-beating after adding 200 mg of 0.1 mm zirconia/silica beads (Thistle Scientific, Glasgow, UK) on a Qiagen TissueLyser II (Qiagen, Manchester, UK) for 5 minutes at 25 Hz. Next, 200 μl of 100% ethanol was added, the sample was centrifuged, the swab head was discarded, and the pellet was purified in four subsequent centrifugation steps after adding one-by-one 200 μl 100% ethanol, 500 μl buffer AW1, 500 μl buffer AW2 and 75 μl buffer AE as per manufacturer’s instructions (Qiagen, Manchester, UK). We included one negative control (an empty tube) with each DNA extraction round of 24 study samples to be able to detect contaminants in extraction reagents downstream. The DNA concentration of randomly selected samples was measured by Qubit (Invitrogen, Thermo Scientific, Paisley, UK) and the DNA quality of all samples by Nanodrop (Thermo Scientific, Paisley, UK).

*PCR amplification and 16S rRNA gene sequencing*

Each of the DNA samples (study samples and negative controls) underwent two PCR rounds for 16S rRNA gene amplification and barcoding. In the first PCR round, the V3-V4 region of the 16S rRNA gene was amplified as described previously.^9^ DNA was amplified in a 25 μl reaction volume using 1.25 μl of a 10 µM concentration of 319F 5’-ACTCCTACGGGAGGCAGCAG-3’ forward primer and 1.25 μl of a 10 µM concentration of 806R 5’-GGACTACHVGGGTWTCTAAT-3’ reverse primer, 12.5 μl NEB Next HF 2x PCR Master Mix (New England Biolabs, Hitchin, UK), 9 μl of nuclease-free water and 1 μl of DNA extraction product. The first denaturation cycle took 30 seconds at 98 ^0^C and was followed by 10 cycles consisting of a denaturation cycle of 10 seconds (at 98 ^0^C), an annealing cycle of 30 seconds (at 58 ^0^C), an extension cycle of 30 seconds (at 72 ^0^C), and a final extension cycle of 5 minutes at 72 ^0^C. PCR products were then purified and size-selected using Agencourt AMPure XP beads (Beckman Coulter, High Wycombe, UK) in a 0.8:1.0 bead-to-sample ratio. The second PCR round was to barcode V3-V4 sequences by a dual-index approach using standard Illumina Nextera XT index kit v2 (Illumina, San Diego, CA, USA), permitting multiplexing of up to 384 samples. The barcoding was performed in a 25 μl reaction volume using 2.5 μl of Index 1 primer, 2.5 μl of Index 2 primer, 12.5 μl NEB Next HF 2x PCR Master Mix and 7.5 μl sample. The first denaturation cycle took 3 minutes at 98 ^0^C and was followed by 15 cycles consisting of a denaturation cycle of 30 seconds (at 98 ^0^C), an annealing cycle of 30 seconds (at 55 ^0^C), an extension cycle of 30 seconds (at 72 ^0^C), and a final extension cycle of 5 minutes at 72 ^0^C. PCR products were then purified using AMPure beads as explained above, again in a 0.8:1.0 bead-to-sample ratio. We added a negative control to each PCR run (10 μl of nuclease-free water instead of 9 μl of nuclease-free water and 1 μl of DNA) to identify contaminants, as well as a commercially available positive control (10 μl of 0.2 ng/μl ZymoBiomics Microbial Community DNA standard; Zymo Research Corp, Irvine, CA, USA). The DNA extraction negative controls were also included in the PCR runs. DNA from samples collected at different visits but belonging to the same participant were included in the same PCR run to control for inherent differences between PCR runs. PCR product DNA concentrations of each sample (including negative and positive controls) were measured using the Qubit Fluorometer with the dsDNA HS Assay kit (Invitrogen, Thermo Scientific, Paisley, UK). All samples, including the positive and negative controls, were successfully amplified and used for subsequent steps.

Amplicons from samples were evenly pooled into sequencing libraries at a mass of 0.8 ng DNA per amplicon. To achieve this, Qubit DNA concentrations were used to calculate the volumes of each study sample to be added. Samples with a DNA concentration of <0.30 ng/µl (e.g., the negative controls) were added in a fixed volume of 1 µl. The libraries were sequenced on an Illumina HiSeq instrument (Illumina, San Diego, CA, USA), run in rapid mode, 2x300bp using a 250PE and 50PE kit. DNA from samples collected at different visits but belonging to the same participant were included in the same library to control for inherent differences between sequencing runs.

*Panbacterial 16S rRNA gene qPCR*

The panbacterial 16S rRNA gene copy concentrations of all samples collected at study visits and containing at least 1,111 reads by Illumina HiSeq sequencing (N=134; see ‘Further data processing’ below for rationale) were determined at the Institute for Genome Sciences of the University of Maryland (Baltimore, MD, USA) using the BactQuant qPCR assay. This assay was developed based on an analyses of 4,938 16S rRNA gene sequences in the Greengenes database.^10,11^ The DNA samples were tested as described previously.^11,12^ Briefly, 1.5 μl of template (1:10 diluted DNA) was added to 3.5 μl of reaction mix, with the final reaction containing 1.8 μM each of the forward (341F) and reverse (806R) primer targeting the 16S V3-V4 region, 225 nM of the TaqManW probe, 1X Platinum Quantitative PCR SuperMix-UDG with ROX (Invitrogen, Thermo Scientific, Waltham, MA, USA) and molecular-grade water. Each experiment included an in-run standard curve (ranging from 10 to 10^8^, with 10^2^–10^8^ in 10-fold serial linear dilutions) and no-template controls performed in triplicate. Amplification and real-time fluorescence detections were performed on the Bio-Rad CFX 384 instrument (Bio-Rad Inc., Hercules, CA, USA) using the following PCR conditions: 3 minutes at 50 °C for UDG treatment, 10 minutes at 95 °C for Taq activation, 15 seconds at 95 °C for denaturation and 1 minute at 60 °C for annealing and extension, times 40 cycles. Cycle threshold (Ct) value for each 16S qPCR reaction were obtained using a manual Ct threshold of 0.05 and automatic baseline. The 16S rRNA gene concentration was reported in copies/μL for each sample.

*Molecular data processing*

We obtained a mean raw unpaired read count of 307,969 reads per study sample (95% confidence interval (CI): 289,284 – 326,654 reads). Reads were first demultiplexed, and primer sequences were removed from forward and reverse reads using Cutadapt 1.2.1.^13^ All subsequent steps were performed in the DADA2 version 1.4.0 package for large paired end datasets in R version 3.4.3 (R core team, 2015).^14^ We chose DADA2 because of its ability to resolve reads differing by only one nucleotide. Error correction was performed using the *fastqFilter* command with parameter settings aiming to maximize read retention. For forward and reverse reads, respectively, the minimum read lengths (*truncLen*) were set to 260 and 210 based on the quality plots, *maxEE* to a maximum of 5 and 8 expected errors, *maxN* to zero ambiguous bases allowed, and *truncQ* to zero. Around 10% of reads were discarded after error correction. Next, error rates of forward and reverse reads were determined using the *learnErrors* command. Forward and reverse reads were dereplicated (assigned to unique amplicon sequence variants (ASVs)) using the *derepFastq* command, and denoised (ASVs with higher than average error rates discarded) using the *dada* command.^14,15^ Forward and reverse reads were then merged into overlapping reads using the *mergePairs* command. Bimeras (chimeric compositions of two separate parent ASVs) were removed using the *removeBimeraDenovo* command with the Silva version 128 database as the reference database;^16^ 10.1% of ASVs were identified as bimeric and removed. Overall, a median of 16% of the raw reads per study sample was removed during these DADA2 clean-up steps.

Taxonomic assignment was also done in DADA2 in two steps: *assignTaxonomy* to map ASVs to taxa at genus level or above using the RDP classifier with a minimum bootstrap value of 50% and the Silva v128 database as the reference database,^16,17^ followed by *addSpecies* to map ASVs to species level, allowing only ASVs with exact (100%) identity matches with species in the Silva database to be assigned to that species, and allowing assignment of one ASV to multiple species.

*Further data processing*

Further data processing was performed in Microsoft Excel 2013 (starting with a spreadsheet containing the sequences, taxonomic assignments, and read counts for each ASV per sample) and STATA version 13 (StataCorp, College Station, TX, USA). We removed all rare ASVs (defined as a read count in all samples combined of less than 100), four non-bacterial ASVs, and two likely contaminant ASVs (a *Rhodanobacter glycinis/terrae* and a *Sneathia* genus) that were present in more than one negative control at relative abundances higher than in any study sample. The vaginal taxa BV-associated bacterium 1 (BVAB1), BVAB2, *Mageeibacillus indolicus* (BVAB3), BVAB TM7 and *Fenollaria massiliensis* are not included in the Silva v128 database but their sequences have been published elsewhere. We identified all of the above species in our ASV spreadsheet using the *Needleman-Wunsch Global Align Nucleotide Sequences* function on the National Center for Biotechnology Information (NCBI) website,^18^ requiring 100% matches between reference sequences and uploaded DADA2-derived ASVs of interest. The Silva-based taxonomic assignment of 146 ASVs with a relative abundance of at least 0.05% of the read count of all samples combined (out of a total of 1,797 ASVs) were double-checked using the *Microbial Nucleotide BLAST (BLASTn)* function on the NCBI website.^19,20^ Using the non-redundant V3-V4 version of the Vaginal 16S rDNA Reference Database as a tiebreaker,^21^ three discordances were resolved, 24 *Lactobacillus* genus ASVs were reassigned to various *Lactobacillus* species, six *Gardnerella* genus ASVs were reassigned to *G. vaginalis*, and one *Atopobium* genus ASV was reassigned to *A. vaginae*. Next, read counts for ASVs assigned to the exact same taxonomy were summed for each sample. Finally, we rarefied at a depth of 1,111 reads (the lowest total read count for a specific sample above 1,000 reads) using the *GUniFrac* 1.0 package in R.^22^ The rarefied ASV table contained 134 samples and 204 unique ASVs (2/136 samples became invalid due to rarefaction), with 133 (65.2%) mapping to species level, 55 (27.0%) to genus level, and 16 (7.8%) to higher taxonomic levels. Rarefied read counts were transformed into relative abundances using the *prop.table* function in R. Of the 204 ASVs, 108 ASVs were present at a relative abundance of at least 1% in at least one sample; the other 96 ASVs were minority species.

*Panbacterial 16S rRNA gene data*

Of the 134 samples that were tested by BactQuant assay, five samples did not amplify in two of three, or all three, of the triplicate reactions, or had skewed low 16S rRNA gene concentration results of <1,000 copies/μl and were considered outliers. A total of 5/134 samples (= 3.7%) were therefore excluded from all concentration analyses. We estimated the ASV-specific concentrations per sample using the sample-specific 16S rRNA gene concentration data. First, we identified the 16S rDNA gene copy number per ASV in the NCBI version of the rrnDB database,^23^ and in the case of missing data, in the RDP version of the rrnDB database (which only contains information at genus level and above) and the Greengenes database.^10^ If an ASV was mapped to multiple species at genus level, the mean of the mean 16S gene copy number for each individual species was used. If the mean 16S gene copy number of a species was not present in the database, we used the mean copy number of the corresponding genus. BVAB1 and BVAB2 belong to the *Clostridiales* order and lower level taxonomic information is not available. We therefore used the *Clostridiales* order copy number (=4.62). Concentrations in cells/μl per ASV per sample were estimated by multiplying the ASV-specific copy-normalized rarefied relative abundance by the sample-specific 16S rRNA gene copies concentration. This method has been shown by others to correlate well with species-specific quantitative PCR results for non-minority species.^24,25^ It yielded concentrations for 204 ASVs in 129 samples in cells/μl, which were log_10_-transformed. Concentrations between zero and one cell/μl were set to one prior to log_10_-transformation to prevent skewed negative values.

*VMB bacterial groups and VMB types*

These are described in the main manuscript. We provide one example here for further clarification. A sample containing 30% *L. iners*, 30% other lactobacilli, 20% *G. vaginalis*, and 20% other BV anaerobes, would have been assigned to the LA (lactobacilli and anaerobes) VMB type. If this sample contained one million 16S rRNA genes per μl, and each of the species included in the sample only contained one 16S rRNA gene copy, it would contain a lactobacilli concentration of [30%+30%] x one million = 600,000/μl, a BV-anaerobes concentration of [20%+20%] x one million = 400,000/μl, a pathobionts concentration of 0/μl, and a ‘other bacteria’ concentration of 0/μl. All VMB types, except for the two *Lactobacillus*-dominated VMB types, were considered dysbiotic. The bacterial groups BV-anaerobes and pathobionts were also considered dysbiotic.

*Statistical analysis and figures*

Concentration changes were expressed as percentages per individual participant as follows: [concentration at the post-treatment visit] – [concentration at the pre-treatment visit] divided by [concentration at the pre-treatment visit]. When the pre-treatment concentration was zero and the post-treatment concentration was greater than zero, the increase was set to 100% or the highest value among the other participants, whichever was greatest.

Bar graphs, bar charts, and scatter plots were made in STATA. Other data visualizations were made in R: three-dimensional plots of the three main non-metric multidimensional scaling (NMDS) vectors were made using *vegan* and *plotly* packages,^26,27^ heatmaps showing the twenty ASVs with highest median relative abundance were made using the *gplots* package,^28^ and alluvial diagrams were made using the *ggalluvial* package to compare (pooled) VMB types before and after treatment.^29^

*Informed consent procedures and reimbursement*

Non-married participants aged 18-20 provided signed consent of a parent/guardian to participate, per Rwandan law at the time of the study. The participants received the equivalent of 3 GBP in local currency as reimbursement for their time spent at the clinic and transport costs. Care was taken to protect participant privacy and confidentiality.

**Appendix A references**

1. Ferris DG, Litaker MS, Woodward L, Mathis D, Hendrich J. Treatment of bacterial vaginosis: a comparison of oral metronidazole, metronidazole vaginal gel, and clindamycin vaginal cream. *J Fam Pract*. 1995;41(5):443-449.

2. Paavonen J, Mangioni C, Martin MA, Wajszczuk CP. Vaginal clindamycin and oral metronidazole for bacterial vaginosis: a randomized trial. *Obstet Gynecol*. 2000;96(2):256-260. doi:10.1016/S0029-7844(00)00902-9

3. van de Wijgert JHHM. The vaginal microbiome and sexually transmitted infections are interlinked: consequences for treatment and prevention. *PLOS Med*. 2017;14(12):e1002478. doi:10.1371/journal.pmed.1002478

4. Cuylaerts V, De Baetselier I, Muvunyi CM, et al. Implementation and evaluation of the Presto combined qualitative real-time assay for *Chlamydia trachomatis* and *Neisseria gonorrhoeae* in Rwanda. *Afr J Lab Med*. 2019;8(1):739. doi:10.4102/ajlm.v8i1.739

5. van de Wijgert JHHM, Borgdorff H, Verhelst R, et al. The vaginal microbiota: what have we learned after a decade of molecular characterization? *PLOS ONE*. 2014;9(8):e105998. doi:10.1371/journal.pone.0105998

6. van de Wijgert JH, Jespers V. Incorporating microbiota data into epidemiologic models: examples from vaginal microbiota research. *Ann Epidemiol*. 2016;26(5):360-365. doi:10.1016/j.annepidem.2016.03.004

7. van de Wijgert JHHM, Jespers V. The global health impact of vaginal dysbiosis. *Res Microbiol*. 2017;168(9):859-864. doi:10.1016/j.resmic.2017.02.003

8. Gill C, van de Wijgert JHHM, Blow F, Darby AC. Evaluation of lysis methods for the extraction of bacterial DNA for analysis of the vaginal microbiota. *PLOS ONE*. 2016;11(9):e0163148. doi:10.1371/journal.pone.0163148

9. Fadrosh DW, Ma B, Gajer P, et al. An improved dual-indexing approach for multiplexed 16S rRNA gene sequencing on the Illumina MiSeq platform. *Microbiome*. 2014;2(1):6. doi:10.1186/2049-2618-2-6

10. DeSantis TZ, Hugenholtz P, Larsen N, et al. Greengenes, a chimera-checked 16S rRNA gene database and workbench compatible with ARB. *Appl Environ Microbiol*. 2006;72(7):5069-5072. doi:10.1128/AEM.03006-05

11. Liu CM, Aziz M, Kachur S, et al. BactQuant: an enhanced broad-coverage bacterial quantitative real-time PCR assay. *BMC Microbiol*. 2012;12:56. doi:10.1186/1471-2180-12-56

12. Nowak RG, Randis TM, Desai P, et al. Higher levels of a cytotoxic protein, vaginolysin, in *Lactobacillus*-deficient community state types at the vaginal mucosa. *Sex Transm Dis*. December 2017:1. doi:10.1097/OLQ.0000000000000774

13. Martin M. Cutadapt removes adapter sequences from high-throughput sequencing reads. *EMBnet.journal*. 2011;17(1):10-12.

14. Callahan BJ, McMurdie PJ, Rosen MJ, Han AW, Johnson AJA, Holmes SP. DADA2: high resolution sample inference from Illumina amplicon data. *Nat Methods*. 2016;13(7):581-583. doi:10.1038/nmeth.3869

15. Rosen MJ, Callahan BJ, Fisher DS, Holmes SP. Denoising PCR-amplified metagenome data. *BMC Bioinformatics*. 2012;13:283. doi:10.1186/1471-2105-13-283

16. Pruesse E, Quast C, Knittel K, et al. SILVA: a comprehensive online resource for quality checked and aligned ribosomal RNA sequence data compatible with ARB. *Nucleic Acids Res*. 2007;35(21):7188-7196. doi:10.1093/nar/gkm864

17. Wang Q, Garrity GM, Tiedje JM, Cole JR. Naïve bayesian classifier for rapid assignment of rRNA sequences into the new bacterial taxonomy. *Appl Environ Microbiol*. 2007;73(16):5261-5267. doi:10.1128/AEM.00062-07

18. Needleman-Wunsch alignment of two nucleotide sequences. https://blast.ncbi.nlm.nih.gov/Blast.cgi?PAGE_TYPE=BlastSearch&PROG_DEF=blastn&BLAST_PROG_DEF=blastn&BLAST_SPEC=GlobalAln&LINK_LOC=BlastHomeLink. Accessed August 15, 2018.

19. Altschul SF, Gish W, Miller W, Myers EW, Lipman DJ. Basic local alignment search tool. *J Mol Biol*. 1990;215(3):403-410. doi:10.1016/S0022-2836(05)80360-2

20. BLAST: Basic Local Alignment Search Tool. https://blast.ncbi.nlm.nih.gov/Blast.cgi. Accessed August 15, 2018.

21. Fettweis JM, Serrano MG, Sheth NU, et al. Species-level classification of the vaginal microbiome. *BMC Genomics*. 2012;13 (Suppl 8):S17. doi:doi: 10.1186/1471-2164-13-S8-S17

22. Chen J, Bittinger K, Charlson ES, et al. Associating microbiome composition with environmental covariates using generalized UniFrac distances. *Bioinformatics*. 2012;28(16):2106-2113. doi:10.1093/bioinformatics/bts342

23. Stoddard SF, Smith BJ, Hein R, Roller BRK, Schmidt TM. rrnDB: improved tools for interpreting rRNA gene abundance in bacteria and archaea and a new foundation for future development. *Nucleic Acids Res*. 2015;43(Database issue):D593-D598. doi:10.1093/nar/gku1201

24. Boshier FAT, Srinivasan S, Fredricks DN, Schiffer JT. Complementing 16S rRNA gene amplicon sequencing with estimates of total bacterial load can infer absolute bacterial species concentrations as measured by targeted assays in the vaginal microbiome. Conference abstract presented at: Keystone Symposia Role of the Genital Tract Microbiome in Sexual and Reproductive Health; 11-15 December 2018; Cape Town, South Africa. Available at: https://www.keystonesymposia.org/views/Web/Meetings/dsp_PrintAllAbstracts.cfm?MeetingID=1660. Accessed 6 March 2019.

25. Jian C, Luukkonen P, Yki-Jarvinen H, Salonen A, Korpela K. Quantitative PCR provides a simple and accessible method for quantitative microbiome profiling: *bioRxiv*. November 2018. doi:10.1101/478685

26. Oksanen J, Blanchet FG, Friendly M, et al. *Vegan: Community Ecology Package*.; 2018. https://CRAN.R-project.org/package=vegan. Accessed August 15, 2018.

27. Sievert C, Parmer C, Hocking T, et al. *Plotly: Create Interactive Web Graphics via “Plotly.Js.”*; 2018. https://CRAN.R-project.org/package=plotly. Accessed August 15, 2018.

28. Warnes GR, Bolker B, Bonebakker L, et al. *Gplots: Various R Programming Tools for Plotting Data*.; 2016. https://CRAN.R-project.org/package=gplots. Accessed September 12, 2018.

29. Brunson JC. *Ggalluvial: Alluvial Diagrams in “Ggplot2.”*; 2018. https://CRAN.R-project.org/package=ggalluvial. Accessed August 15, 2018.

**Fig. A.1: Flowchart of the VMB study**

Abbreviations: *BV* bacterial vaginosis, *M6* Month 6 visit, *RU* Rinda Ubuzima, *STI* sexually transmitted infection, *TV* *Trichomonas vaginalis*, *UTI* urinary tract infection.

Flow chart of the VMB study, including the randomized vaginal microbiota maintenance component that will be reported elsewhere.

^1^ Valid Nugent data available for 67 women; valid rarefied sequencing data available for 67 women; valid qPCR-based estimated concentrations available for 66 women.

^2^ Valid Nugent data available for 66 women; valid rarefied sequencing data available for 67 women; valid qPCR-based estimated concentrations available for 63 women. Valid concentration data for both pre- and post-treatment were available for 61 women.

^3^ Totals to 110 reasons among 102 women because multiple reasons could have been reported by one woman.

^4^ Reasons: outside of metronidazole treatment window (n=5), enrollment target already met (n=4), has a mental disorder (n=1), did not complete screening procedures and was subsequently lost to follow=up (n=1), withdrew consent during the pre-treatment (Screening) visit because she thought the reimbursement was too low (n=1)

^5^ No data are available for these two women; no vaginal swabs were taken as these women did not pass subsequent enrollment procedures.

^6^ Successful treatment was defined as having a Nugent score of 7-10 before treatment and 0-3 after treatment (N=30), while treatment failure was defined as having a Nugent score of 7-10 before treatment and 4-10 after treatment (N=25). Thirteen women were excluded from these analyses because they did not have Nugent 7-10 at the pre-treatment visit (N=12) or did not have a valid Nugent result at the post-treatment visit (N=1).

**Fig. A.2: Alluvial diagrams of vaginal microbiota types pre- and post-treatment**

| **a** | **b** |
| --- | --- |
| 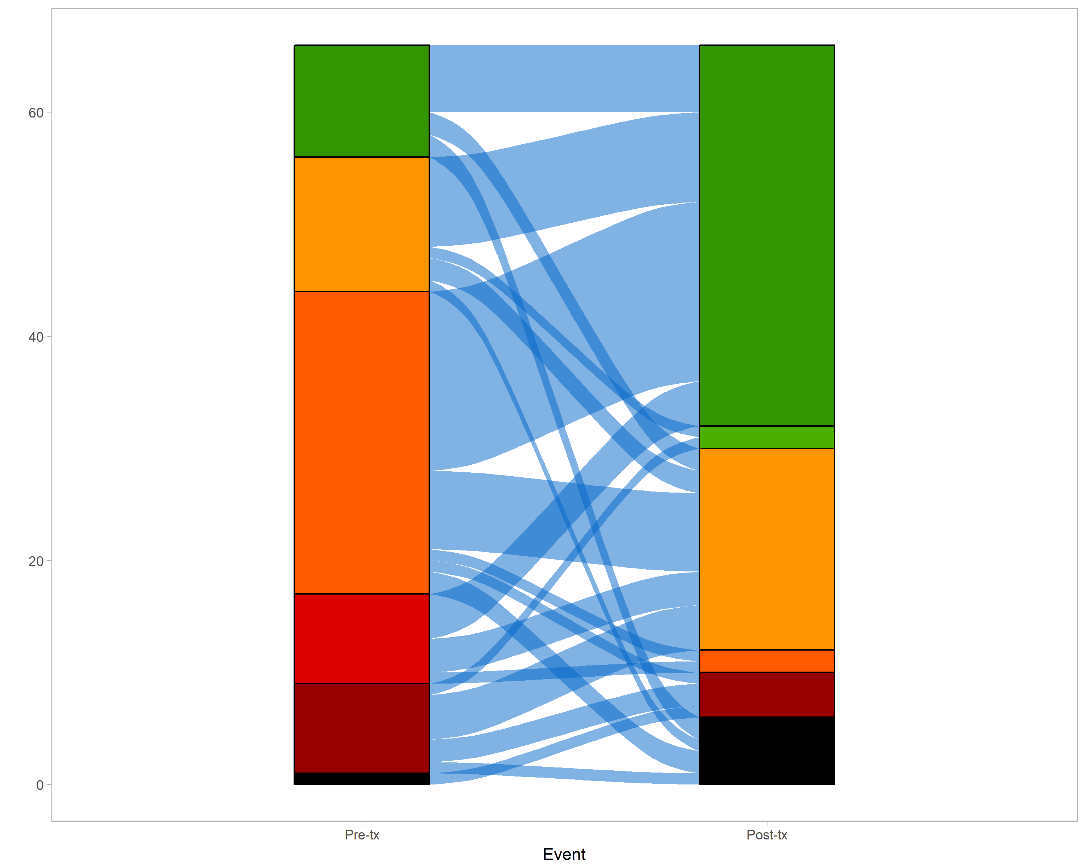 | 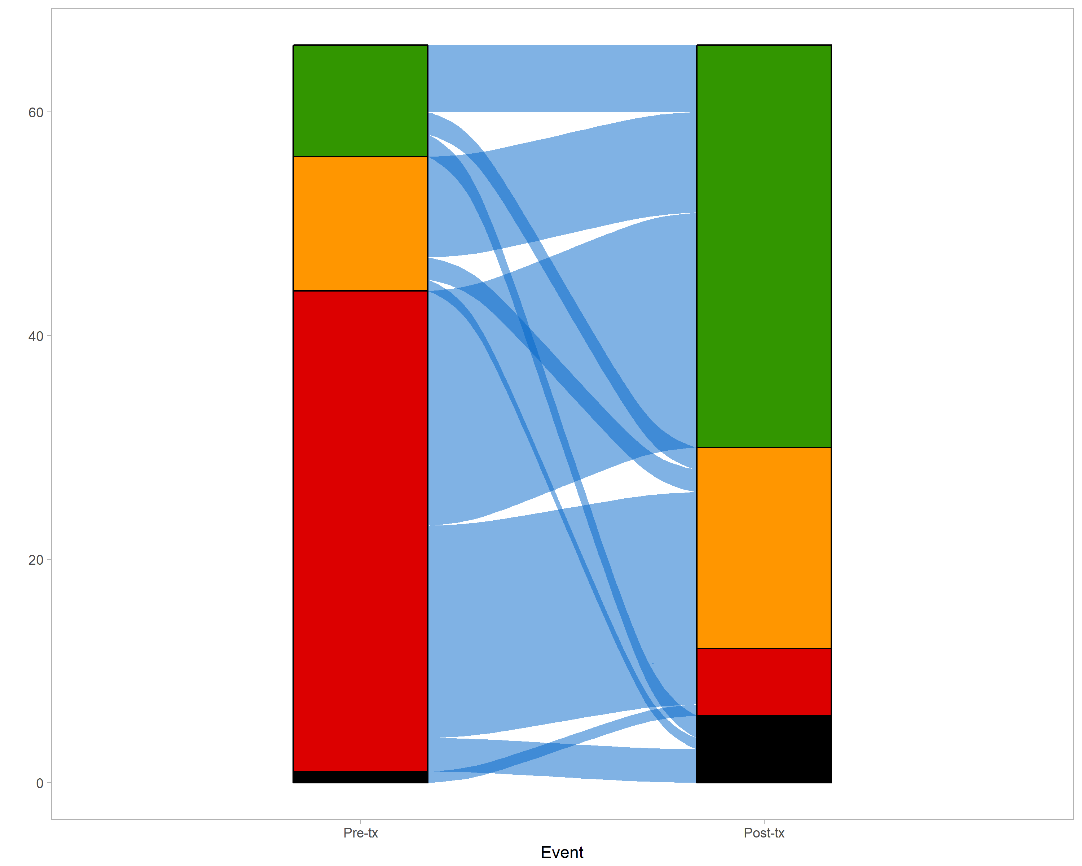 |
| 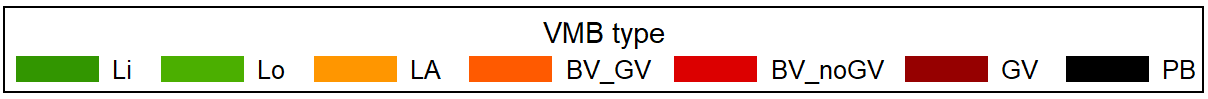 | 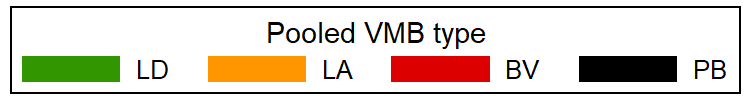 |

Abbreviations: *BV* bacterial vaginosis, *BV_GV* polybacterial *Gardnerella vaginalis*-containing, *BV_noGV* polybacterial but low *G. vaginalis*, *GV* *G. vaginalis*-dominated, *LA* lactobacilli and anaerobes, *LD* *Lactobacillus*-dominated, *Li* *L.* iners-dominated, *Lo* other lactobacilli-dominated, *PB* pathobionts, *Pre-tx* pre-treatment visit, *Post-tx* post-treatment visit, *VMB* vaginal microbiota.

**a** Changes in VMB types (n=66). Two participants with missing VMB types at either the pre-treatment (n=1) or post-treatment (n=1) visit are not shown. **b** Changes in pooled VMB types (n=66). VMB types were pooled into *Lactobacillus*-dominated (LD; combining VMB types Li and Lo), lactobacilli and anaerobes (LA), BV-like (combining VMB types BV_noGV, BV_GV, and GV) and pathobionts (PB).

**Table A.1: Bacterial group concentrations and relative abundances by treatment success**

| **VMB outcomes at pre-treatment visit** | **All participants (n = 66)** | **Successful tx^1^**  **(n = 29)** | **Unsuccessful tx^2^**  **(n = 25)** | **p^3^** |
| --- | --- | --- | --- | --- |
| Mean total bacterial concentration in log_10_/μL (95% CI)^4^ | 6.59  (6.39 – 6.78) | 6.59  (6.31 – 6.86) | 6.68  (6.36 – 7.01) | 0.656 |
| Mean total *Lactobacillus* concentration in log_10_/μL (95% CI)^4^ | 4.98  (4.61 – 5.35) | 4.92  (4.36 – 5.49) | 4.62  (3.92 – 5.31) | 0.605 |
| Mean total BV-anaerobes concentration in log_10_/μL (95% CI)^4^ | 6.23  (5.88 – 6.57) | 6.46  (6.14 – 6.78) | 6.62  (6.26 – 6.97) | 0.557 |
| Mean total pathobionts concentration in log_10_/μL (95% CI)^4^ | 1.85  (1.29 – 2.41) | 1.09  (0.32 – 1.87) | 2.30  (1.40 – 3.19) | 0.037 |
| Mean total other bacteria concentration in log_10_/μL (95% CI)^4^ | 3.33  (2.84 – 3.83) | 1.71  (0.96 – 2.45) | 2.44  (1.56 – 3.31) | 0.132 |
| Mean RA total *Lactobacillus* (95% CI) | 0.24  (0.15 – 0.32) | 0.18  (0.08 – 0.27) | 0.10  (0.02 – 0.18) | 0.410 |
| Mean RA total BV-anaerobes (95% CI) | 0.74  (0.66 – 0.82) | 0.81  (0.71 – 0.91) | 0.89  (0.81 – 0.97) | 0.263 |
| Mean RA total pathobionts (95% CI) | 0.02  (0.01 – 0.03) | 0.01  (0.00 – 0.02) | 0.01  (0.00 – 0.03) | 0.079 |
| Mean RA total other bacteria (95% CI) | 0.01  (0 – 0.01) | 0  (0 – 0) | 0  (0 – 0) | 0.099 |
| **Changes in VMB outcomes (comparing differences between visits)** | **All participants (n = 66)** | **Successful tx^1^**  **(n = 28)** | **Unsuccessful tx^2^**  **(n = 25)** | **p^3^** |
| Difference in mean total bacterial concentration in log_10_/μL (95% CI)^4^ | -0.71  (-0.95 – -0.48) | -0.96  (-1.29 – -0.64) | -0.44  (-0.82 – -0.06) | 0.026 |
| Difference in mean total *Lactobacillus* concentration in log_10_/μL (95% CI)^4^ | 0.47  (0.11 – 0.83) | 0.45  (-0.01 – 0.92) | 1.06  (0.37 – 1.75) | 0.095 |
| Difference in mean total BV-anaerobes concentration in log_10_/μL (95% CI)^4^ | -1.61  (-2.07 – -1.15) | -2.68  (-3.28 – -2.09) | -0.81  (-1.32 – -0.31) | <0.001 |
| Difference in mean total pathobionts concentration in log_10_/μL (95% CI)^4^ | 0.09  (-0.56 – 0.74) | 0.24  (-0.51 – 0.98) | 0.29  (-1.06 – 1.64) | 0.790 |
| Difference in mean total other bacteria concentration in log_10_/μL (95% CI)^4^ | -1.63  (-2.24 – -1.03) | -0.79  (-1.62– 0.04) | -0.25  (-1.36– -0.86) | 0.403 |
| Difference in mean RA total *Lactobacillus* (95% CI) | 0.47  (0.37 – 0.57) | 0.69  (0.55 – 0.83) | 0.47  (0.34 – 0.59) | 0.002 |
| Difference in mean RA total BV-anaerobes bacteria (95% CI) | -0.51  (-0.60 – -0.41) | -0.73  (-0.85 – -0.61) | -0.48  (-0.61 – -0.35) | 0.001 |
| Difference in mean RA total pathobionts  (95% CI) | 0.04  (0.00 – 0.07) | 0.04  (-0.02 – 0.10) | 0.02  (-0.01 – 0.05) | 0.689 |
| Difference in mean RA total other bacteria (95% CI) | 0  (0 – 0) | 0  (-0.01 – 0) | 0  (-0.01 – 0) | 0.457 |

Abbreviations: *BV* bacterial vaginosis, *CI* confidence interval, *RA* relative abundance, *tx* treatment, *VMB* vaginal microbiota.

^1^ Defined as having a Nugent score of 7-10 prior to treatment and 0-3 after treatment.

^2^ Defined as having a Nugent score of 7-10 prior to treatment and 4-10 after treatment.

^3^ By Mann-Whitney U test, comparing those with successful treatment to those with unsuccessful treatment.

^4^ Concentration data may contain at most five missing values due to invalid results.

**Table A.2: VMB characteristics pre- and post-treatment, stratified by ongoing CT/NG infection**

| **VMB Outcomes** | **All participants** | | | **CT/NG negative at baseline^2^** | | | **CT/NG positive at baseline^2^** | | |
| --- | --- | --- | --- | --- | --- | --- | --- | --- | --- |
|  | **Pre-treatment**  **(n = 68)** | **Post-treatment**  **(n = 68)** | **p^1^** | **Pre-treatment**  **(n = 41)** | **Post-treatment**  **(n = 41)** | **p^1^** | **Pre-treatment**  **(n = 26)** | **Post-treatment**  **(n = 26)** | **p^1^** |
| Nugent categories (n %)^3^   - 0-3 - 4-6 - 7-10 | 5 (7.5)  6 (9.0)  56 (83.6) | 36 (54.6)  13 (19.7)  17 (25.8) | <0.001 | 5 (12.2)  3 (7.2)  33 (80.5) | 24 (58.5)  6 (14.6)  11 (26.8) | <0.001 | 0  3 (11.5)  33 (88.5) | 12 (48.0)  7 (28.0)  6 (24.0) | 0.001 |
| Mean inverse Simpson diversity index (95% CI)^4^ | 0.67  (0.60 – 0.73) | 0.31  (0.25 – 0.38) | <0.001 | 0.63  (0.53 – 0.72) | 0.30  (0.21 – 0.39) | <0.001 | 0.74  (0.65 – 0.82) | 0.33  (0.22 – 0.45) | <0.001 |
| VMB type (n %)^4^:  – Li   - Lo - LA - BV_GV - BV_noGV - GV - PB | 10 (14.9)  0  12 (17.9)  28 (41.8)  8 (11.9)  8 (11.9)  1 (1.5) | 35 (52.2)  2 (3.0)  18 (26.9)  2 (3.0)  0  4 (6.0)  6 (9.0) | <0.001 | 9 (22.0)  0  10 (24.4)  15 (36.6)  3 (7.3)  4 (9.8)  0 | 20 (48.8)  1 (2.4)  13 (31.7)  1 (2.4)  0  2 (4.9)  4 (9.8) | 0.001 | 1 (3.9)  0  2 (7.7)  13 (50.0)  5 (19.2)  4 (15.4)  1 (3.9) | 15 (57.7)  1 (3.9)  5 (19.2)  1 (3.9)  0  2 (7.7)  2 (7.7) | 0.003 |
| Vaginal pH, median (IQR) | 5.3  (5.0 – 5.6) | 4.4  (3.6 – 4.6) | <0.001 | 5.3  (4.7 – 5.6) | 4.3  (3.6 – 4.4) | <0.001 | 5.5  (5.0 – 5.6) | 4.4  (3.6 – 4.7) | <0.001 |
| Vulvovaginal candidiasis (n %) | 6 (8.8) | 4 (5.9) | 0.527 | 5 (11.9) | 2 (4.8) | 0.257 | 1 (3.9) | 2 (7.7) | 0.564 |
| **Bacterial group relative abundances: mean (95% CI)**^4^ | | | | | | | | | |
| Total lactobacilli | 0.24  (0.15 – 0.32) | 0.72  (0.64 – 0.80) | <0.001 | 0.32  (0.20 – 0.43) | 0.73  (0.63 – 0.83) | <0.001 | 0.10  (0.02 – 0.19) | 0.70  (0.56 – 0.84) | <0.001 |
| Total BV-associated anaerobes | 0.75  (0.67 – 0.83) | 0.23  (0.16 – 0.30) | <0.001 | 0.67  (0.55 – 0.79) | 0.23  (0.14 – 0.33) | <0.001 | 0.87  (0.79 – 0.96) | 0.23  (0.11 – 0.34) | <0.001 |
| Total pathobionts | 0.02  (0.01 – 0.03) | 0.05  (0.02 – 0.09) | 0.050 | 0.01  (0 – 0.02) | 0.04  (0.01 – 0.07) | 0.821 | 0.02  (0 – 0.05) | 0.07  (-0.01 – 0.15) | 0.015 |
| Total other bacteria | 0  (0 – 0) | 0  (0 – 0) | 0.674 | 0  (0 – 0) | 0  (0 – 0) | 0.354 | 0  (0 – 0) | 0  (0 – 0.01) | 0.590 |
| **Bacterial group concentrations in log_10_ cells/μL: mean (95% CI)^5^** | | | | | | | | | |
| Total bacteria | 6.59  (6.39 – 6.78) | 5.85  (5.66 – 6.04) | <0.001 | 6.44  (6.20 – 6.68) | 5.90  (5.66 – 6.13) | 0.002 | 6.82  (6.49 – 7.14) | 5.78  (5.44 – 6.11) | <0.001 |
| Total lactobacilli | 4.98  (4.61 – 5.35) | 5.56  (5.34 – 5.78) | 0.017 | 5.02  (4.52 – 5.52) | 5.63  (5.39 – 5.88) | 0.112 | 4.92  (4.33 – 5.50) | 5.43  (4.99 – 5.88) | 0.072 |
| Total BV-anaerobes | 6.23  (5.88 – 6.57) | 4.55  (4.14 – 4.95) | <0.001 | 5.89  (5.40 – 6.39) | 4.44  (3.87 – 5.01) | <0.001 | 6.73  (6.36 – 7.09) | 4.73  (4.17 – 5.28) | <0.001 |
| Total pathobionts | 1.92  (1.36 – 2.48) | 2.01  (1.48 – 2.54) | 0.939 | 1.59  (0.91 – 2.27) | 1.65  (0.96 – 2.32) | 0.464 | 2.43  (1.43 – 3.42) | 2.60  (1.73 – 3.47) | 0.474 |
| Total other bacteria | 1.85  (1.36 – 2.35) | 1.46  (1.01 – 1.92) | 0.176 | 1.68  (1.06 – 2.30) | 1.29  (0.74 – 1.84) | 0.216 | 2.12  (1.27 – 2.97) | 1.74  (0.90 – 2.58) | 0.554 |
| **Individual bacterial species/genera concentrations in log_10_ cells/μL: mean (95% CI)^5^** | | | | | | | | | |
| *L. iners* | 4.81  (4.38 – 5.24) | 5.28  (4.94 – 5.62) | 0.072 | 4.89  (4.32 – 5.46) | 5.33  (4.89 – 5.76) | 0.394 | 4.69  (4.00 – 5.39) | 5.21  (4.63 – 5.80) | 0.072 |
| *L. crispatus*^6^ | 0.15  (-0.02 – 0.33) | 0.51  (0.16 – 0.85) | 0.089 | 0.25  (-0.04 – 0.55) | 0.54  (0.06 – 1.02) | 0.388 | 0  (0 – 0) | 0.45  (-0.07 – 0.97) | 0.084 |
| Other lactobacilli^7^ | 1.46  (0.97 – 1.94) | 3.03  (2.57 – 3.48) | <0.001 | 1.74  (1.11 – 2.37) | 3.17  (2.60 – 3.74) | 0.002 | 1.03  (0.23 – 1.82) | 2.79  (1.99 – 3.85) | 0.002 |
| *Gardnerella vaginalis* | 5.62  (5.20 – 6.03) | 4.12  (3.63 – 4.61) | <0.001 | 5.23  (4.60 – 5.86) | 3.96  (3.27 – 4.65) | 0.009 | 6.22  (5.85 – 6.59) | 4.37  (3.68 – 5.07) | <0.001 |
| *Atopobium vaginae* | 4.58  (4.00 – 5.16) | 1.54  (1.06 – 2.02) | <0.001 | 4.13  (3.31 – 4.94) | 1.73  (1.10 – 2.37) | <0.001 | 5.27  (4.50 – 6.03) | 1.22  (0.45 – 1.99) | <0.001 |
| *Prevotella* species | 4.67  (4.18 – 5.16) | 1.35  (0.90 – 1.79) | <0.001 | 4.29  (3.60 – 4.98) | 1.41  (0.85 – 1.98) | <0.001 | 5.25  (4.60 – 5.90) | 1.24  (0.46 – 2.01) | <0.001 |
| *Sneathia* species | 4.18  (3.63 – 4.73) | 1.08  (0.63 – 1.54) | <0.001 | 3.79  (3.05 – 4.52) | 0.85  (0.28 – 1.43) | <0.001 | 4.79  (3.97 – 5.61) | 1.44  (0.66 – 2.21) | <0.001 |
| *Megasphaera* species | 3.17  (2.56 – 3.79) | 0.22  (-0.01 – 0.44) | <0.001 | 2.74  (1.91 – 3.56) | 0.13  (-0.06 – 0.33) | <0.001 | 3.84  (2.92 – 4.76) | 0.36  (-0.16 – 0.88) | <0.001 |
| *Veillonella* species | 2.37  (1.75 – 3.00) | 0.28  (0.01 – 0.56) | <0.001 | 2.22  (1.46 – 2.99) | 0.45  (0.01 – 0.99) | 0.001 | 2.60  (1.46 – 3.73) | 0  (0 – 0) | 0.001 |
| BVAB1 | 1.76  (1.11 – 2.42) | 0.46  (0.15 – 0.77) | <0.001 | 0.99  (0.29 – 1.69) | 0.24  (0.01 – 0.47) | 0.072 | 2.95  (1.77 – 4.13) | 0.83  (0.09 – 1.57) | 0.002 |
| *Fusobacterium* species | 0.53  (0.17 – 0.89) | 0  (0 – 0) | 0.008 | 0.33  (-0.05 – 0,70) | 0  (0 – 0) | 0.083 | 0.85  (0.12 – 1.57) | 0  (0 – 0) | 0.046 |
| *Streptococcus* species | 1.47  (0.92 – 2.02) | 1.34  (0.84 – 1.85) | 0.453 | 1.50  (0.82 – 2.17) | 1.14  (0.53 – 1.75) | 0.056 | 1.43  (0.44 – 2.43) | 1.67  (0.73 – 2.60) | 0.350 |
| *Staphylococcus* species | 0.26  (0.05 – 0.47) | 0.60  (0.27 – 0.93) | 0.655 | 0.29  (0.01 – 0.57) | 0.31  (0 – 0.61) | 0.317 | 0.22  (-0.10 – 0.53) | 1.07  (0.36 – 1.79) | 0.317 |
| *Escherichia*/*Shigella* | 0.10  (-0.04 – 0.25) | 0.86  (0.45 – 1.27) | 0.317 | 0.17  (-0.07 – 0.41) | 0.70  (0.20 – 1.20) | 0.317 | 0  (0 – 0) | 1.12  (0.39 – 1.85) | Not determinable |

Abbreviations: *BV* bacterial vaginosis, *BVAB1* BV-associated bacterium type 1, *BV_GV* polybacterial *Gardnerella vaginalis*-containing, *BV_noGV* polybacterial low *G. vaginalis*, *CI* confidence interval, *CT* *Chlamydia trachomatis, GV* *G. vaginalis*-dominated, *LA* lactobacilli and anaerobes, *Li* *L. iners*-dominated, *Lo* other lactobacilli-dominated, *NG* *Neisseria gonorrhoeae, PB* pathobionts-containing, *VMB* vaginal microbiota.

^1^ Stuart-Maxwell test for matched categorical data, and Wilcoxon signed-rank test for matched continuous data.

^2^ Participants included regardless of Nugent score at pre-treatment and post-treatment visits.

^3^ Valid Nugent data available for 67 participants at the pre-treatment visit and 66 participants at the post-treatment visit.

^4^ Relative abundance, Simpson inverse diversity indices, and VMB type data available for 67 participants at each visit.

^5^  Concentration data may contain at most five missing values (see Appendix A Supplementary Methods).

^6^  Includes all amplicon sequence variants attributed to *L. crispatus*, also those with multiple species assignments.

^7^  Includes amplicon sequence variants attributed to *L. jensenii*, *L. delbrueckii*, *L. fermentum*, *L. gasseri*, *L. johnsonii*, and *Lactobacillus genus*, as well as 11 other minority amplicon sequence variants.

**Table A.3: VMB characteristics pre- and post-treatment, stratified by additional antibiotic use**

| **VMB Outcomes** | **All participants** | | | **Used metronidazole only^2^** | | | **Used metronidazole plus another antibiotic^2^** | | |
| --- | --- | --- | --- | --- | --- | --- | --- | --- | --- |
|  | **Pre-treatment**  **(n = 68)** | **Post-treatment**  **(n = 68)** | **p^1^** | **Pre-treatment**  **(n = 50)** | **Post-treatment**  **(n = 50)** | **p^1^** | **Pre-treatment**  **(n = 18)** | **Post-treatment**  **(n = 18)** | **p^1^** |
| Nugent categories (n %)^3^   - 0-3 - 4-6 - 7-10 | 5 (7.5)  6 (9.0)  56 (83.6) | 36 (54.6)  13 (19.7)  17 (25.8) | <0.001 | 3 (6.1)  5 (10.2)  41 (83.7) | 27 (55.1)  8 (16.3)  14 (28.6) | <0.001 | 2 (11.1)  1 (5.6)  15 (83.8) | 9 (52.9)  5 (29.4)  3 (17.7) | 0.005 |
| Mean inverse Simpson diversity index (95% CI)^4^ | 0.67  (0.60 – 0.73) | 0.31  (0.25 – 0.38) | <0.001 | 0.68  (0.61 – 0.75) | 0.32  (0.24 – 0.40) | <0.001 | 0.64  (0.49 – 0.79) | 0.29  (0.15 – 0.43) | 0.005 |
| VMB type (n %)^4^:  – Li   - Lo - LA - BV_GV - BV_noGV - GV - PB | 10 (14.9)  0  12 (17.9)  28 (41.8)  8 (11.9)  8 (11.9)  1 (1.5) | 35 (52.2)  2 (3.0)  18 (26.9)  2 (3.0)  0  4 (6.0)  6 (9.0) | <0.001 | 7 (14.3)  0  9 (18.4)  19 (38.8)  7 (14.3)  6 (12.2)  1 (2.0) | 26 (62.0)  2 (4.0)  13 (26.0)  2 (4.0)  0  3 (6.0)  4 (8.0) | <0.001 | 3 (16.7)  0  3 (16.7)  9 (50.0)  1 (5.6)  2 (11.1)  0 | 9 (52.9)  0  5 (29.4)  0  0  1 (5.9)  2 (11.8) | 0.054 |
| Vaginal pH, median (IQR) | 5.3  (5.0 – 5.6) | 4.4  (3.6 – 4.6) | <0.001 | 5.3  (5.0 – 5.6) | 4.4  (3.6 – 5.6) | <0.001 | 5.5  (5.0 – 5.6) | 4.4  (4.1 – 4.7) | <0.001 |
| Vulvovaginal candidiasis (n %) | 6 (8.8) | 4 (5.9) | 0.527 | 3 (6.0) | 4 (8.0) | 0.706 | 3 (16.7) | 0 | 0.083 |
| **Bacterial group relative abundances: mean (95% CI)**^4^ | | | | | | | | | |
| Total lactobacilli | 0.24  (0.15 – 0.32) | 0.72  (0.64 – 0.80) | <0.001 | 0.22  (0.13 – 0.32) | 0.71  (0.62 – 0.81) | <0.001 | 0.26  (0.08 – 0.45) | 0.72  (0.55 – 0.89) | 0.006 |
| Total BV-associated anaerobes | 0.75  (0.67 – 0.83) | 0.23  (0.16 – 0.30) | <0.001 | 0.75  (0.66 – 0.85) | 0.23 (0.15 – 0.32) | <0.001 | 0.73  (0.54 – 0.91) | 0.22  (0.07 – 0.37) | 0.001 |
| Total pathobionts | 0.02  (0.01 – 0.03) | 0.05  (0.02 – 0.09) | 0.050 | 0.02  (0.01 – 0.04) | 0.05  (0.01 – 0.09) | 0.218 | 0  (0 – 0.01) | 0.05  (-0.01 – 0.11) | 0.092 |
| Total other bacteria | 0  (0 – 0) | 0  (0 – 0) | 0.674 | 0  (0 – 0) | 0  (0 – 0) | 0.323 | 0  (0 – 0_ | 0.01  (0 – 0.01) | 0.416 |
| **Bacterial group concentrations in log_10_ cells/μL: mean (95% CI)^5^** | | | | | | | | | |
| Total bacteria | 6.59  (6.39 – 6.78) | 5.85  (5.66 – 6.04) | <0.001 | 6.66  (6.44 – 6.88) | 5.86  (5.64 – 6.08) | <0.001 | 6.40  (5.96 – 6.84) | 5.83  (5.41 – 6.25) | 0.114 |
| Total lactobacilli | 4.98  (4.61 – 5.35) | 5.56  (5.34 – 5.78) | 0.017 | 4.99  (4.51 – 5.47) | 5.54  (5.29 – 5.80) | 0.087 | 4.95  (4.42 – 5.48) | 5.60  (5.09 – 6.10) | 0.109 |
| Total BV-anaerobes | 6.23  (5.88 – 6.57) | 4.55  (4.14 – 4.95) | <0.001 | 6.29  (5.89 – 6.69) | 4.54  (4.04 – 5.03) | <0.001 | 6.05  (5.34 – 6.77) | 4.59  (3.86 – 5.31) | 0.008 |
| Total pathobionts | 1.92  (1.36 – 2.48) | 2.01  (1.48 – 2.54) | 0.939 | 2.02  (1.35 – 2.70) | 1.99 (1.36 – 2.63) | 0.725 | 1.65  (0.54 – 2.75) | 2.06  (0.98 – 3.14) | 0.545 |
| Total other bacteria | 1.85  (1.36 – 2.35) | 1.46  (1.01 – 1.92) | 0.176 | 1.99  (1.41 – 2.57) | 1.39  (0.88 – 1.90) | 0.093 | 1.49  (0.50 – 2.49) | 1.67  (0.59 – 2.76) | 0.704 |
| **Individual bacterial species/genera concentrations in log_10_ cells/μL: mean (95% CI)^5^** | | | | | | | | | |
| *L. iners* | 4.81  (4.38 – 5.24) | 5.28  (4.94 – 5.62) | 0.072 | 4.84  (4.31 – 5.37) | 5.25  (4.84 – 5.65) | 0.291 | 4.74  (3.96 – 5.51) | 5.39  (4.71 – 6.08) | 0.109 |
| *L. crispatus*^6^ | 0.15  (-0.02 – 0.33) | 0.51  (0.16 – 0.85) | 0.089 | 0.13  (-0.06 – 0.32) | 0.37  (0 – 0.75) | 0.180 | 0.21  (-0.24 – 0.67) | 0.90 (0.03 – 1.76) | 0.271 |
| Other lactobacilli^7^ | 1.46  (0.97 – 1.94) | 3.03  (2.57 – 3.48) | <0.001 | 1.40  (0.82 – 1.98) | 2.92  (2.38 – 3.47) | <0.001 | 1.18  (0.45 – 1.91) | 3.31  (2.45 – 4.17) | 0.046 |
| *Gardnerella vaginalis* | 5.62  (5.20 – 6.03) | 4.12  (3.63 – 4.61) | <0.001 | 5.65  (5.15 – 6.16) | 4.18 (3.61 – 4.74) | <0.001 | 5.52  (4.71 – 6.34) | 3.95  (2.83 – 5.06) | 0.070 |
| *Atopobium vaginae* | 4.58  (4.00 – 5.16) | 1.54  (1.06 – 2.02) | <0.001 | 4.52  (3.80 – 5.24) | 1.87  (1.29 – 2.46) | <0.001 | 4.74  (3.72 – 5.76) | 0.55  (-0.11 – 1.21) | 0.001 |
| *Prevotella* species | 4.67  (4.18 – 5.16) | 1.35  (0.90 – 1.79) | <0.001 | 4.74  (4.20 – 5.29) | 1.31  (0.77 – 1.85) | <0.001 | 4.48  (3.29 – 5.66) | 1.45  (0.59 – 2.30) | 0.001 |
| *Sneathia* species | 4.18  (3.63 – 4.73) | 1.08  (0.63 – 1.54) | <0.001 | 4.05  (3.37 – 4.73) | 1.17  (0.61 – 1.73) | <0.001 | 4.51  (3.54 – 5.48) | 0.81  (0.02 – 1.61) | <0.001 |
| *Megasphaera* species | 3.17  (2.56 – 3.79) | 0.22  (-0.01 – 0.44) | <0.001 | 3.23  (2.50 – 3.96) | 0.26  (-0.04 – 0.55) | <0.001 | 3.02  (1.75 – 4.29) | 0.11  (-0.12 – 0.34) | 0.004 |
| *Veillonella* species | 2.37  (1.75 – 3.00) | 0.28  (0.01 – 0.56) | <0.001 | 2.37  (1.63 – 3.12) | 0.38  (0.01 – 0.75) | <0.001 | 2.36  (1.08 – 3.64) | 0  (0 – 0) | 0.005 |
| BVAB1 | 1.76  (1.11 – 2.42) | 0.46  (0.15 – 0.77) | <0.001 | 1.97  (1.17 – 2.78) | 0.56  (0.16 – 0.96) | 0.001 | 1.20  (0.05 – 2.36) | 0.17  (-0.19 – 0.52) | 0.144 |
| *Fusobacterium* species | 0.53  (0.17 – 0.89) | 0  (0 – 0) | 0.008 | 0.47  (0.06 – 0.89) | 0  (0 – 0) | 0.046 | 0.69  (-0.10 – 1.48) | 0  (0 – 0) | 0.084 |
| *Streptococcus* species | 1.47  (0.92 – 2.02) | 1.34  (0.84 – 1.85) | 0.453 | 1.64  (0.96 – 2.31) | 1.47  (0.85 – 2.09) | 0.420 | 1.04  (0.03 – 2.04) | 0.95  (0.07 – 1.84) | 0.911 |
| *Staphylococcus* species | 0.26  (0.05 – 0.47) | 0.60  (0.27 – 0.93) | 0.655 | 0.18  (-0.03 – 0.39) | 0.41  (0.07 – 0.76) | 0.317 | 0.47  (-0.07 – 1.00) | 1.14  (0.29 – 1.99) | 0.317 |
| *Escherichia*/*Shigella* | 0.10  (-0.04 – 0.25) | 0.86  (0.45 – 1.27) | 0.317 | 0.14  (-0.06 – 0.34) | 0.74  (0.27 – 1.20) | 0.317 | 0  (0 – 0) | 1.21  (0.28 – 2.13) | Not determinable |

Abbreviations: *BV* bacterial vaginosis, *BVAB1* BV-associated bacterium type 1, *BV_GV* polybacterial *Gardnerella vaginalis*-containing, *BV_noGV* polybacterial but low *G. vaginalis*, *CI* confidence interval*, GV* *G. vaginalis*-dominated, *LA* lactobacilli and anaerobes, *Li* *L. iners*-dominated, *Lo* other lactobacilli-dominated, *PB* pathobionts-containing, *VMB* vaginal microbiota.

^1^ Stuart-Maxwell test for matched categorical data, and Wilcoxon signed-rank test for matched continuous data.

^2^ The other antibiotic were ciprofloxacin for urinary tract infection and penicillin for syphilis. Both groups include three women each (total n=6) who received antifungal treatment for vulvovaginal candidiasis.

^3^ Valid Nugent data available for 67 participants at the pre-treatment visit and 66 participants at the post-treatment visit.

^4^ Relative abundance, Simpson inverse diversity indices, and VMB type data available for 67 participants at each visit.

^5^  Concentration data may contain at most five missing values (see Appendix A Supplementary Methods).

^6^  Includes all amplicon sequence variants attributed to *L. crispatus*, also those with multiple species assignments.

^7^  Includes amplicon sequence variants attributed to *L. jensenii*, *L. delbrueckii*, *L. fermentum*, *L. gasseri*, *L. johnsonii*, and *Lactobacillus genus*, as well as 11 other minority amplicon sequence variants.

**Table A.4: VMB characteristics pre- and post-treatment, stratified by reported vaginal discharge symptoms at the pre-treatment visit**

| **VMB Outcomes** | **All participants** | | | **Reported vaginal discharge**  **at pre-treatment visit^2^** | | | **Dit not report any vaginal discharge at pre-treatment visit^2^** | | |
| --- | --- | --- | --- | --- | --- | --- | --- | --- | --- |
|  | **Pre-treatment**  **(n = 68)** | **Post-treatment**  **(n = 68)** | **p^1^** | **Pre-treatment**  **(n = 13)** | **Post-treatment**  **(n = 13)** | **p^1^** | **Pre-treatment**  **(n = 55)** | **Post-treatment**  **(n = 55)** | **p^1^** |
| Nugent categories (n %)^3^   - 0-3 - 4-6 - 7-10 | 5 (7.5)  6 (9.0)  56 (83.6) | 36 (54.6)  13 (19.7)  17 (25.8) | <0.001 | 0  2 (16.7)  10 (83.3) | 7 (53.9)  2 (15.4)  4 (30.8) | 0.030 | 5 (9.1)  4 (7.3)  46 (83.6) | 29 (54.7)  11 (20.8)  13 (24.5) | <0.001 |
| Mean inverse Simpson diversity index (95% CI)^4^ | 0.67  (0.60 – 0.73) | 0.31  (0.25 – 0.38) | <0.001 | 0.70  (0.54 – 0.86) | 0.24  (0.08 – 0.39) | 0.006 | 0.66  (0.59 – 0.74) | 0.33  (0.26 – 0.41) | <0.001 |
| VMB type (n %)^4^:  – Li   - Lo - LA - BV_GV - BV_noGV - GV - PB | 10 (14.9)  0  12 (17.9)  28 (41.8)  8 (11.9)  8 (11.9)  1 (1.5) | 35 (52.2)  2 (3.0)  18 (26.9)  2 (3.0)  0  4 (6.0)  6 (9.0) | <0.001 | 2 (15.4)  0  2 (15.4)  5 (38.5)  3 (23.1)  0  1 (7.7) | 9 (69.2)  0  3 (23.1)  0  0  1 (7.7)  0 | 0.109 | 8 (14.8)  0  10 (18.5)  23 (42.6)  5 (9.3)  8 (14.8)  0 | 26 (48.2)  2 (3.7)  15 (27.8)  2 (3.7)  0  3 (5.6)  6 (11.1) | <0.001 |
| Vaginal pH, median (IQR) | 5.3  (5.0 – 5.6) | 4.4  (3.6 – 4.6) | <0.001 | 5.3  (5.3 – 5.6) | 4.1  (3.6 – 4.4) | 0.001 | 5.3  (4.7 – 5.6) | 4.4  (4.1 – 4.7) | <0.001 |
| Vulvovaginal candidiasis (n %) | 6 (8.8) | 4 (5.9) | 0.527 | 0 | 1 (7.7) | 0.317 | 6 (10.9) | 3 (5.5) | 0.317 |
| **Bacterial group relative abundances: mean (95% CI)**^4^ | | | | | | | | | |
| Total lactobacilli | 0.24  (0.15 – 0.32) | 0.72  (0.64 – 0.80) | <0.001 | 0.23  (0.03 – 0.43) | 0.77  (0.58 – 0.96) | 0.005 | 0.24  (0.14 – 0.33) | 0.70  (0.62 – 0.79) | <0.001 |
| Total BV-associated anaerobes | 0.75  (0.67 – 0.83) | 0.23  (0.16 – 0.30) | <0.001 | 0.73  (0.54 – 0.93) | 0.22  (0.03 – 0.41) | 0.007 | 0.75  (0.66 – 0.84) | 0.23  (0.15 – 0.31) | <0.001 |
| Total pathobionts | 0.02  (0.01 – 0.03) | 0.05  (0.02 – 0.09) | 0.050 | 0.04  (-0.01 – 0.08) | 0.01  (0 – 0.02) | 0.597 | 0.01  (0 – 0.02) | 0.06  (0.02 – 0.10) | 0.015 |
| Total other bacteria | 0  (0 – 0) | 0  (0 – 0) | 0.674 | 0  (0 – 0) | 0  (0 – 0) | 0.405 | 0  (0 – 0) | 0  (0 – 0) | 0.386 |
| **Bacterial group concentrations in log_10_ cells/μL: mean (95% CI)^5^** | | | | | | | | | |
| Total bacteria | 6.59  (6.39 – 6.78) | 5.85  (5.66 – 6.04) | <0.001 | 6.47 (6.17 – 6.77) | 5.69  (5.43 – 5.95) | 0.003 | 6.62  (6.38 – 6.85) | 5.89  (5.66 – 6.12) | <0.001 |
| Total lactobacilli | 4.98  (4.61 – 5.35) | 5.56  (5.34 – 5.78) | 0.017 | 5.19 (4.70 – 5.69) | 5.41  (5.03 – 5.79) | 0.433 | 4.93  (4.48 – 5.38) | 5.59  (5.33 – 5.86) | 0.026 |
| Total BV-anaerobes | 6.23  (5.88 – 6.57) | 4.55  (4.14 – 4.95) | <0.001 | 6.25  (5.77 – 6.72) | 4.50  (3.83 – 5.18) | 0.006 | 6.22  (5.81 – 6.63) | 4.56  (4.08 – 5.04) | <0.001 |
| Total pathobionts | 1.92  (1.36 – 2.48) | 2.01  (1.48 – 2.54) | 0.939 | 2.46  (0.96 – 3.95) | 1.75  (0.51 – 2.99) | 0.428 | 1.79 (1.17 – 2.41) | 2.07 (1.46 – 2.68) | 0.618 |
| Total other bacteria | 1.85  (1.36 – 2.35) | 1.46  (1.01 – 1.92) | 0.176 | 1.41  (0.26 – 2.56) | 1.72  (0.68 – 2.75) | 1.00 | 1.96  (1.40 – 2.52) | 1.40  (0.88 – 1.92) | 0.138 |
| **Individual bacterial species/genera concentrations in log_10_ cells/μL: mean (95% CI)^5^** | | | | | | | | | |
| *L. iners* | 4.81  (4.38 – 5.24) | 5.28  (4.94 – 5.62) | 0.072 | 5.14  (4.64 – 5.63) | 4.85  (3.71 – 5.99) | 0.638 | 4.73  (4.21 – 5.26) | 5.39  (5.04 – 5.73) | 0.062 |
| *L. crispatus*^6^ | 0.15  (-0.02 – 0.33) | 0.51  (0.16 – 0.85) | 0.089 | 0  (0 – 0) | 0.43  (-0.51 – 1.36) | 0.317 | 0.19 (-0.03 – 0.41) | 0.53  (0.14 – 0.91) | 0.149 |
| Other lactobacilli^7^ | 1.46  (0.97 – 1.94) | 3.03  (2.57 – 3.48) | <0.001 | 1.02  (-0.17 – 2.21) | 2.32  (1.12 – 3.53) | 0.077 | 1.57  (1.02 – 2.11) | 3.19  (2.70 – 3.69) | <0.001 |
| *Gardnerella vaginalis* | 5.62  (5.20 – 6.03) | 4.12  (3.63 – 4.61) | <0.001 | 5.59  (5.05 – 6.12) | 4.33  (3.62 – 5.05) | 0.023 | 5.62  (5.11 – 6.13) | 4.07  (3.48 – 4.66) | <0.001 |
| *Atopobium vaginae* | 4.58  (4.00 – 5.16) | 1.54  (1.06 – 2.02) | <0.001 | 4.15  (2.64 – 5.65) | 1.53  (0.30 – 2.76) | 0.016 | 4.68  (4.04 – 5.33) | 1.54  (1.00 – 2.08) | <0.001 |
| *Prevotella* species | 4.67  (4.18 – 5.16) | 1.35  (0.90 – 1.79) | <0.001 | 4.87  (4.25 – 5.50) | 1.64  (0.51 – 2.76) | 0.004 | 4.62  (4.02 – 5.22) | 1.28  (0.78 – 1.77) | <0.001 |
| *Sneathia* species | 4.18  (3.63 – 4.73) | 1.08  (0.63 – 1.54) | <0.001 | 4.08  (2.92 – 5.24) | 1.24  (0.01 – 2.47) | 0.008 | 4.20  (3.56 – 4.84) | 1.05  (0.54 – 1.55) | <0.001 |
| *Megasphaera* species | 3.17  (2.56 – 3.79) | 0.22  (-0.01 – 0.44) | <0.001 | 3.22  (1.83 – 4.61) | 0  (0 – 0) | 0.006 | 3.16  (2.45 – 3.87) | 0.27  (-0.01 – 0.55) | <0.001 |
| *Veillonella* species | 2.37  (1.75 – 3.00) | 0.28  (0.01 – 0.56) | <0.001 | 2.17 (0.60 – 3.73) | 0.41  (-0.50 – 1.33) | 0.132 | 2.42  (1.72 – 3.13) | 0.25  (-0.04 – 0.54) | <0.001 |
| BVAB1 | 1.76  (1.11 – 2.42) | 0.46  (0.15 – 0.77) | <0.001 | 2.94 (1.18 – 4.70) | 0.76  (-0.14 – 1.66) | 0.016 | 1.47  (0.77 – 2.17) | 0.39  (0.06 – 0.73) | 0.007 |
| *Fusobacterium* species | 0.53  (0.17 – 0.89) | 0  (0 – 0) | 0.008 | 0.66  (-0.32 – 1.63) | 0  (0 – 0) | 0.158 | 0.50  (0.11 – 0.90) | 0  (0 – 0) | 0.025 |
| *Streptococcus* species | 1.47  (0.92 – 2.02) | 1.34  (0.84 – 1.85) | 0.453 | 2.17  (0.64 – 3.69) | 0.78  (-0.38 – 1.93) | 0.139 | 1.30  (0.71 – 1.90) | 1.47  (0.90 – 2.05) | 0.996 |
| *Staphylococcus* species | 0.26  (0.05 – 0.47) | 0.60  (0.27 – 0.93) | 0.655 | 0.20 (-0.24 – 0.64) | 0.30  (-0.36 – 0.95) | ND | 0.27  (0.04 – 0.51) | 0.67  (0.28 – 1.06) | 0.655 |
| *Escherichia*/*Shigella* | 0.10  (-0.04 – 0.25) | 0.86  (0.45 – 1.27) | 0.317 | 0  (0 – 0) | 0.49  (-0.25 – 1.23) | ND | 0.13  (-0.05 – 0.31) | 0.95  (0.47 – 1.43) | 0.317 |

Abbreviations: *BV* bacterial vaginosis, *BVAB1* BV-associated bacterium type 1, *BV_GV* polybacterial *Gardnerella vaginalis*-containing, *BV_noGV* polybacterial but low *G. vaginalis*, *CI* confidence interval*, GV* *G. vaginalis*-dominated, *LA* lactobacilli and anaerobes, *Li* *L. iners*-dominated, *Lo* other lactobacilli-dominated, *ND* not determinable, *PB* pathobionts-containing, *VMB* vaginal microbiota.

^1^ Stuart-Maxwell test for matched categorical data, and Wilcoxon signed-rank test for matched continuous data.

^2^ Current or in the past two weeks.

^3^ Valid Nugent data available for 67 participants at the pre-treatment visit and 66 participants at the post-treatment visit.

^4^ Relative abundance, Simpson inverse diversity indices, and VMB type data available for 67 participants at each visit.

^5^  Concentration data may contain at most five missing values (see Appendix A Supplementary Methods).

^6^  Includes all amplicon sequence variants attributed to *L. crispatus*, also those with multiple species assignments.

^7^  Includes amplicon sequence variants attributed to *L. jensenii*, *L. delbrueckii*, *L. fermentum*, *L. gasseri*, *L. johnsonii*, and *Lactobacillus genus*, as well as 11 other minority amplicon sequence variants.

**Fig. A.3: VMB outcomes pre- and post-treatment, stratified by metronidazole treatment success**

| **a** | **b** |
| --- | --- |
| **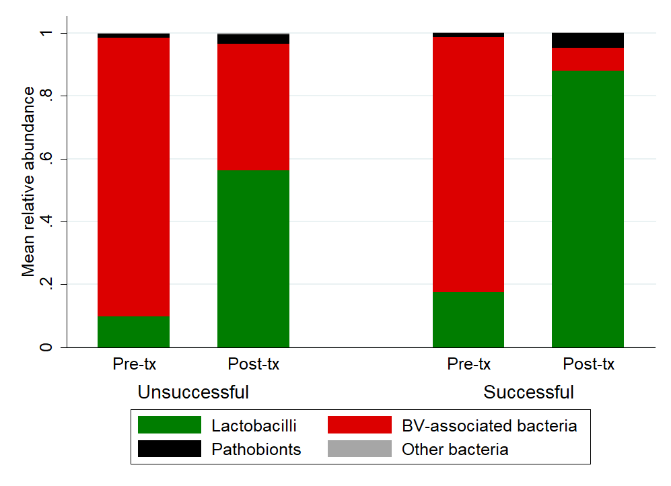** | **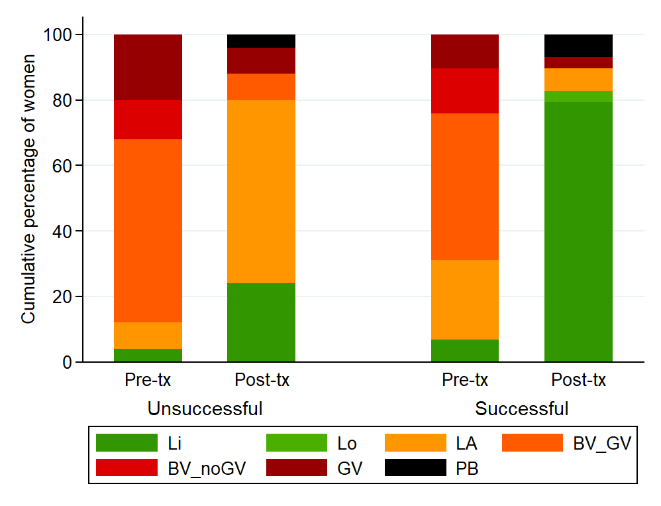** |
| **c** | **d** |
| **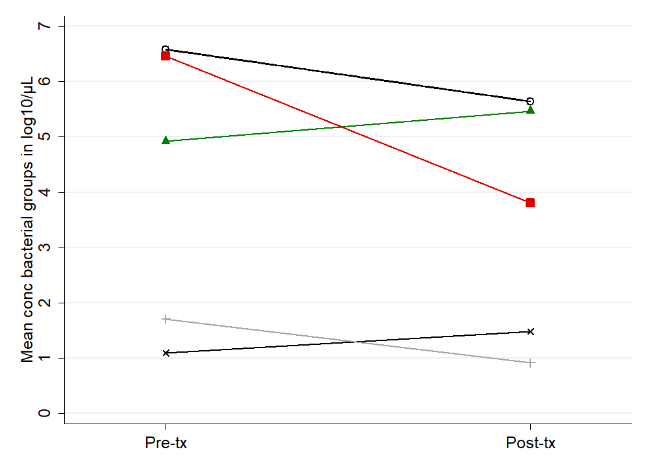** | **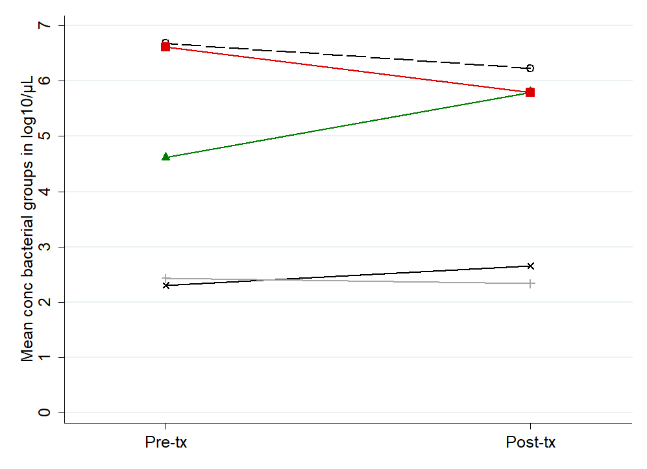** |
| 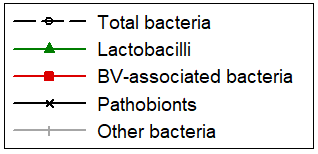 | |
| **e** |  |
| **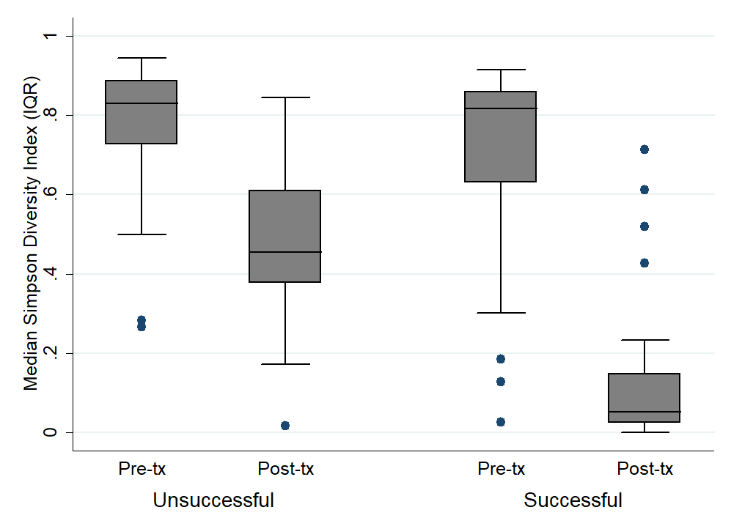** | |

Abbreviations: *BV* bacterial vaginosis, *BV_GV* polybacterial *Gardnerella vaginalis*-containing, *BV_noGV* polybacterial but low *G. vaginalis*, *Conc* concentration, *GV* *G. vaginalis*-dominated, *IQR* inter-quartile range, *LA* lactobacilli and anaerobes, *Li* *L. iners*-dominated, *Lo* other lactobacilli-dominated, *PB* pathobionts-containing, *Pre-tx* pre-treatment visit, *Post-tx* post-treatment visit, *VMB* vaginal microbiota.

**a-e** Figures show changes in VMB characteristics before and after metronidazole treatment: bacterial group mean relative abundances (**a**), VMB types (**b**), bacterial groups concentrations of participants with successful treatment by Nugent scoring (n=28 at pre-tx, n=29 at post-tx; ; see Table 2 for 95% confidence intervals) (**c**). **d** bacterial group concentrations of participants with unsuccessful treatment by Nugent scoring (n=25 at pre-tx, n=22 at post-tx). **e** median inverse Simpson diversity index before and after metronidazole treatment, stratified by treatment success.

**Fig. A.4: VMB outcomes pre- and post-treatment, stratified by ongoing CT/NG infection**

| **a**  **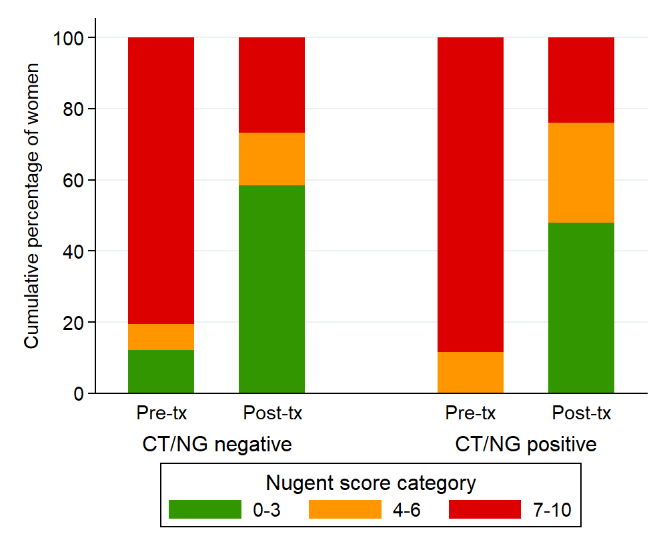** | **b**  **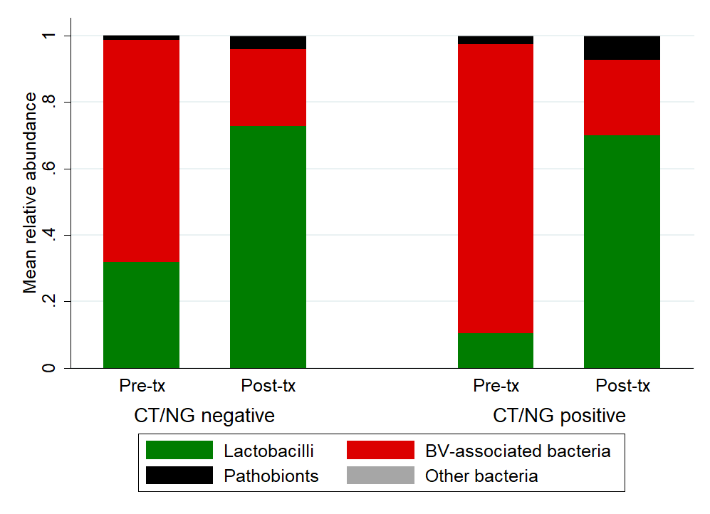** |
| --- | --- |
| **c**  **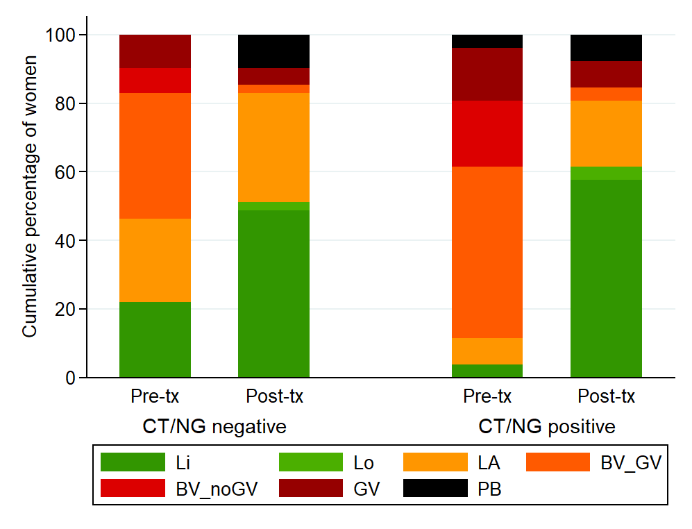** | **d**  **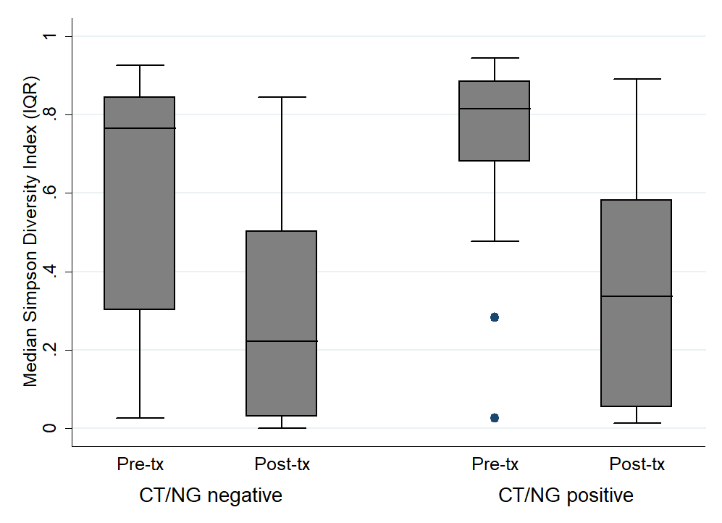** |
| **e**  **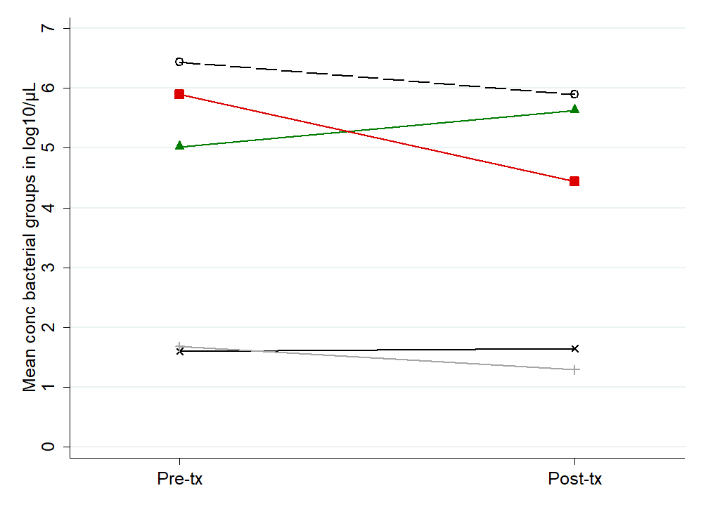** | **f**  **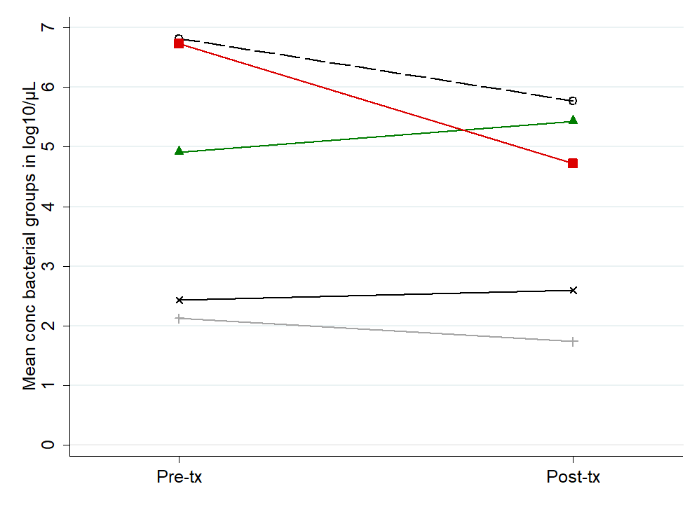** |
| 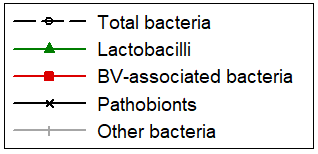 | |

Abbreviations: *BV* bacterial vaginosis, *BV_GV* polybacterial *Gardnerella vaginalis*-containing, *BV_noGV* polybacterial but low *G. vaginalis*, *Conc* concentration, *CT* *Chlamydia trachomatis*, *GV* *G. vaginalis*-dominated, *IQR* inter-quartile range, *LA* lactobacilli and anaerobes, *Li* *L. iners*-dominated, *Lo* other lactobacilli-dominated, *NG* *Neisseria gonorrhoeae*, *PB* pathobionts-containing, *Pre-tx* pre-treatment visit, *Post-tx* post-treatment visit, *VMB* vaginal microbiota.

**a-f** Figures show changes in VMB characteristics before and after metronidazole treatment, stratified by CT/NG status at baseline: Nugent score categories (**a**), bacterial group mean relative abundances (**b**), VMB types (**c**), and median inverse Simpson diversity index (**d**). **e** Bacterial group concentrations of CT/NG-negative participants at baseline (n=40 at pre-tx, n=39 at post-tx; see Table A.2 for 95% confidence intervals). **f** Bacterial group concentrations of CT/NG-positive participants at baseline (n=26 at pre-tx, n=24 at post-tx).

**Fig. A.5: VMB outcomes pre- and post-treatment, stratified by additional antibiotic use**

| **a**  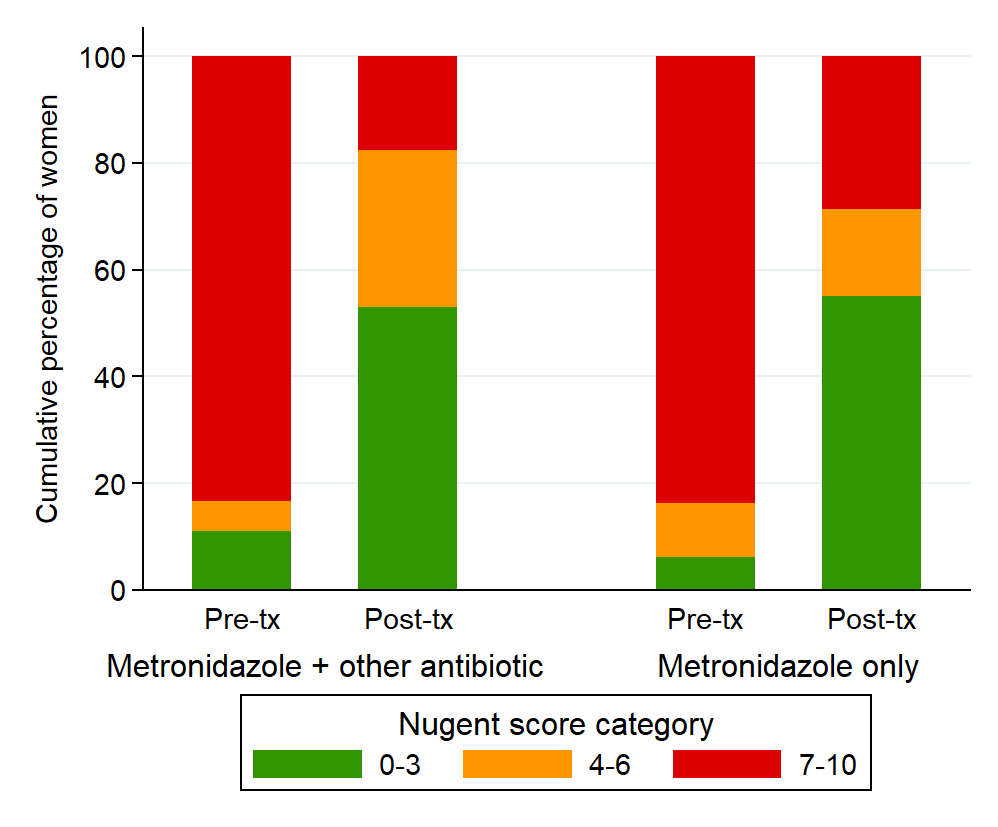 | **b**  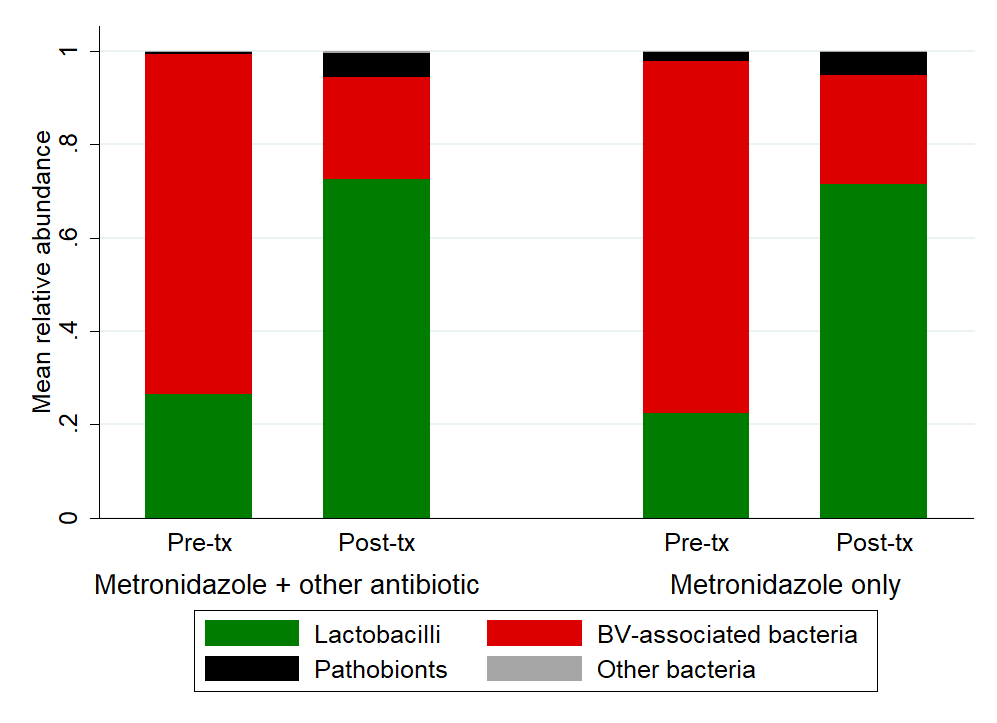 |
| --- | --- |
| **c**  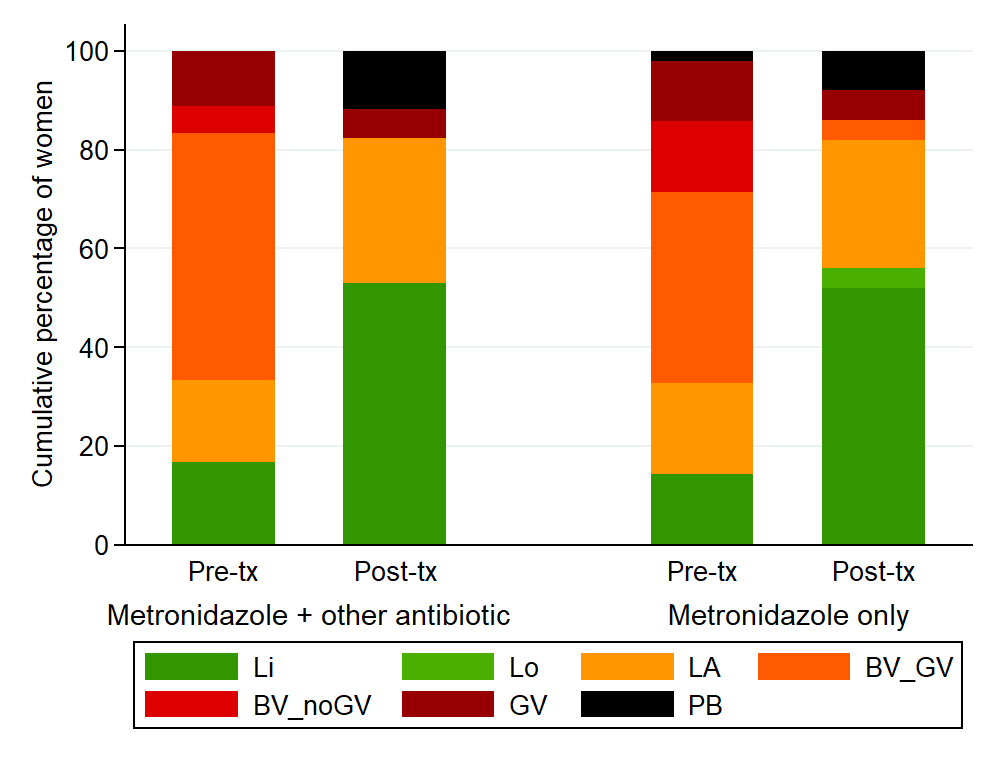 | **d**  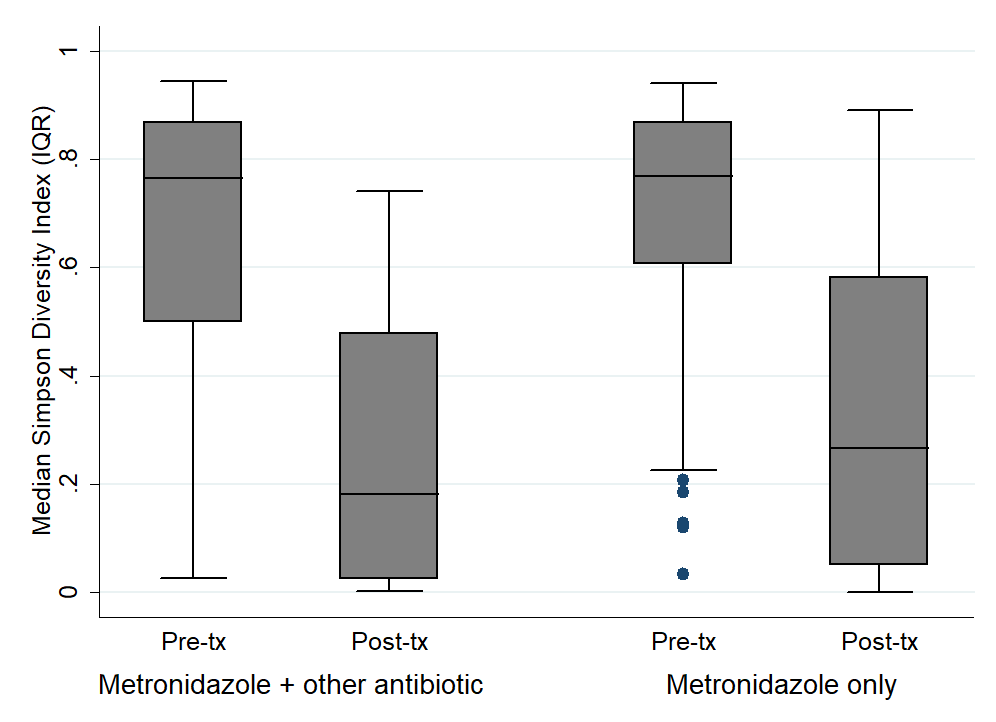 |
| **e**  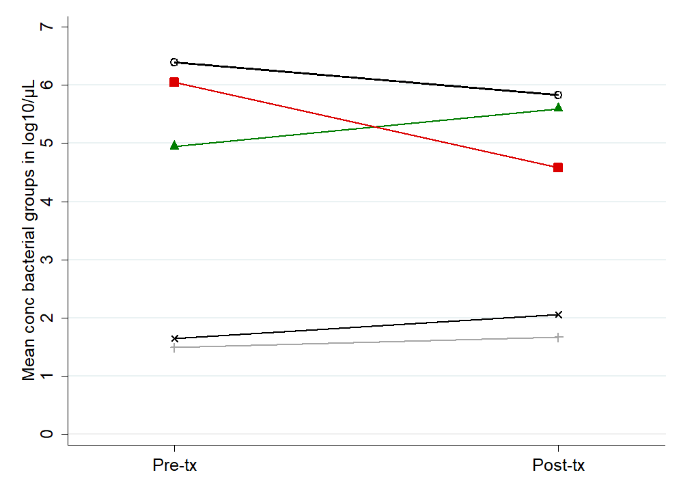 | **f**  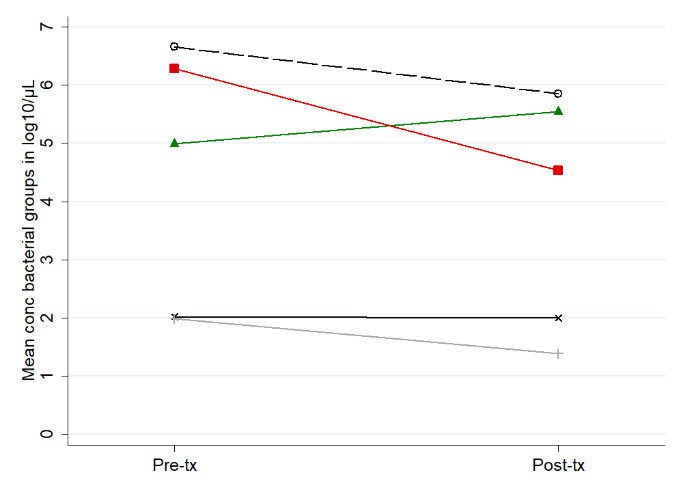 |
| 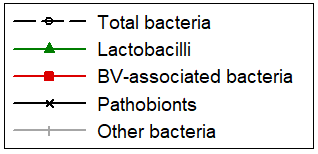 | |

Abbreviations: *BV* bacterial vaginosis, *BV_GV* polybacterial *Gardnerella vaginalis*-containing, *BV_noGV* polybacterial but low *G. vaginalis*, *Conc* concentration, *GV* *G. vaginalis*-dominated, *IQR* inter-quartile range, *LA* lactobacilli and anaerobes, *Li* *L. iners*-dominated, *Lo* other lactobacilli-dominated, *PB* pathobionts-containing, *Pre-tx* pre-treatment visit, *Post-tx* post-treatment visit, *VMB* vaginal microbiota.

**a-f** Figures show changes in VMB characteristics before and after metronidazole treatment, stratified by use of another antibiotic in addition to metronidazole: Nugent score categories (**a**), bacterial group mean relative abundances (**b**), VMB types (**c**), and median inverse Simpson diversity index (**d**). **e** Bacterial group concentrations of participants who received another antibiotic in addition to metronidazole at baseline (n=18 at pre-treatment visit, n=17 at post-treatment visit; see Table A.3 for 95% confidence intervals). **f** Bacterial group concentrations of participants who only received metronidazole at baseline (n=49 at pre-treatment visit, n=50 at post-treatment visit).

**Fig. A.6: VMB outcomes pre- and post-treatment, stratified by pre-treatment vaginal discharge**

| **a**  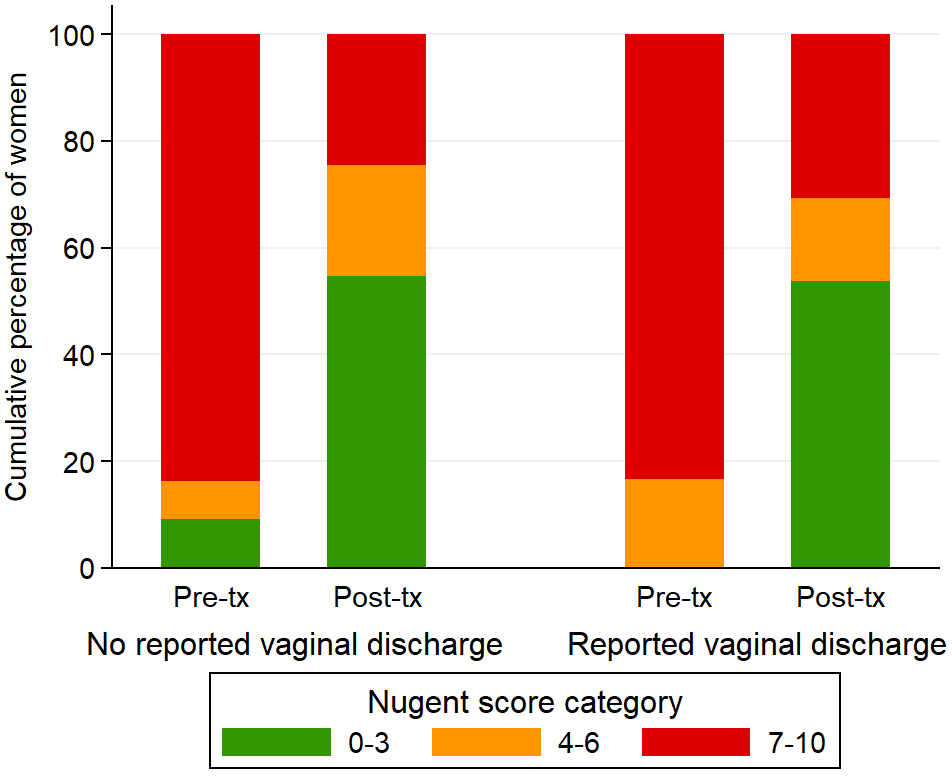 | **b**  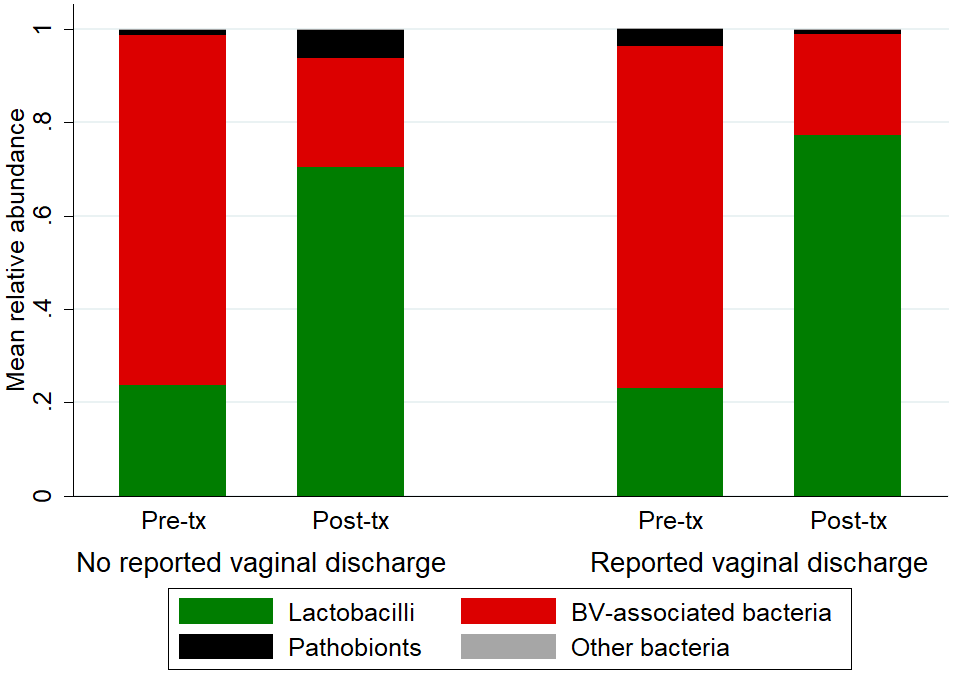 |
| --- | --- |
| **c**  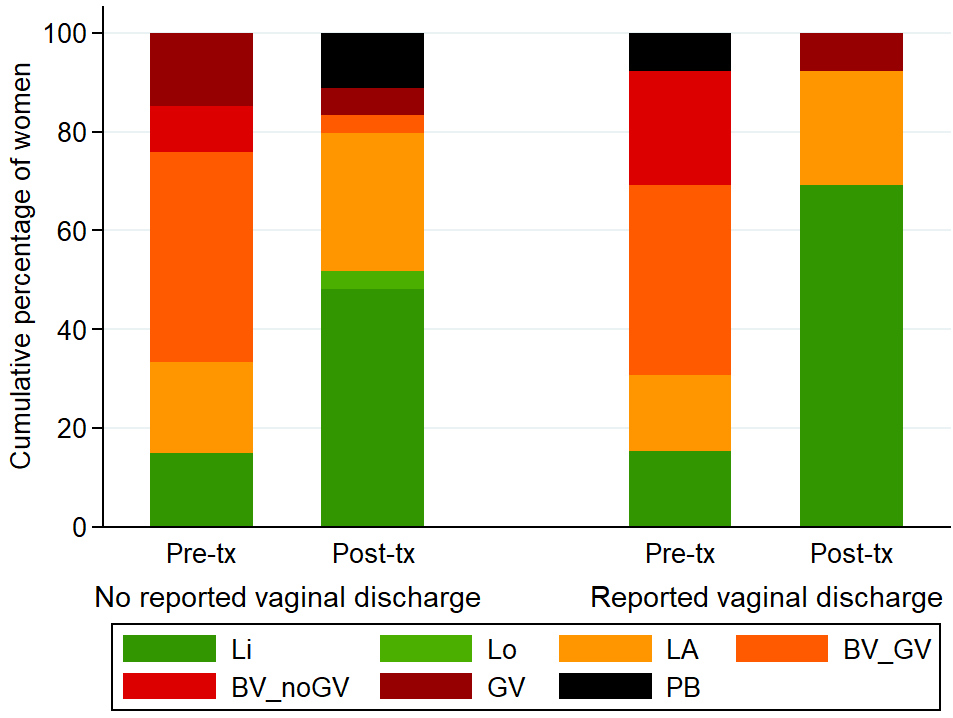 | **d**  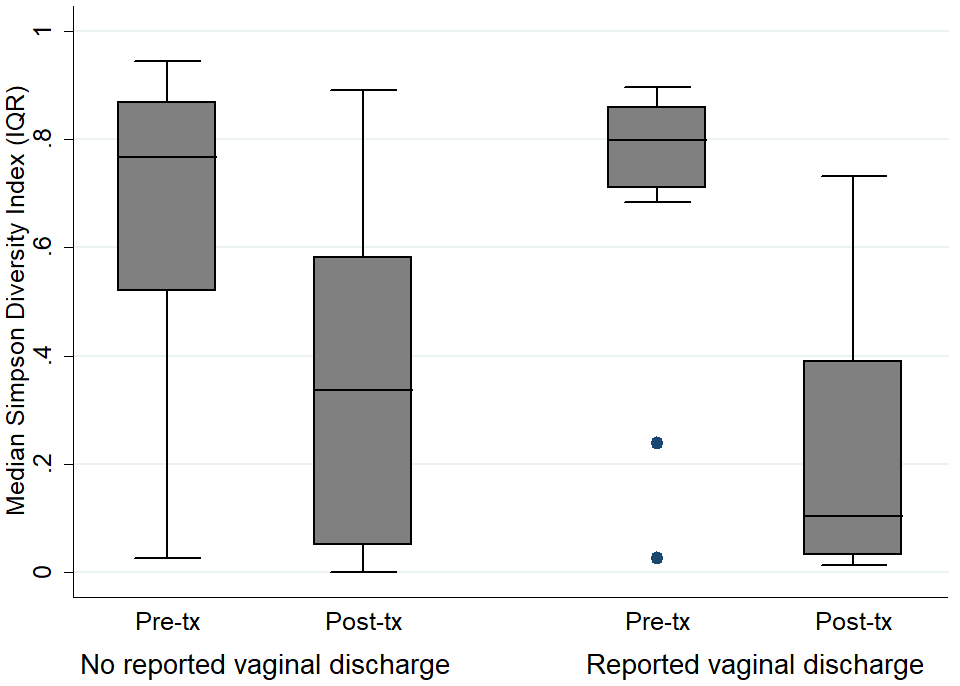 |
| **e**  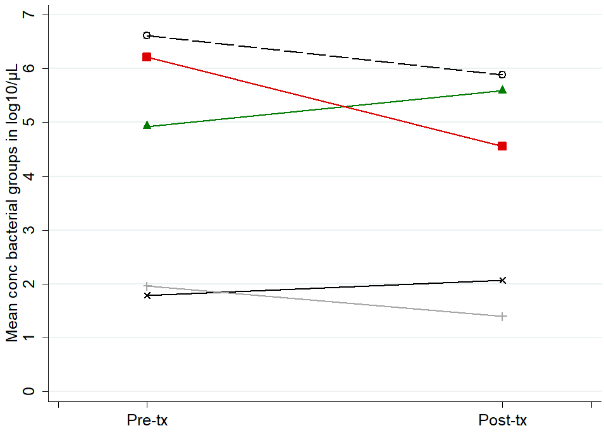 | **f**  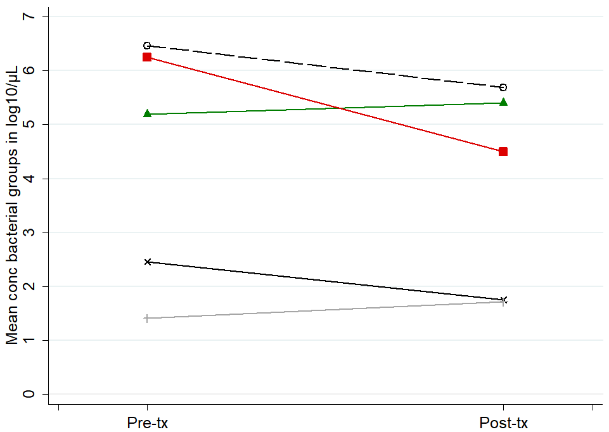 |
| 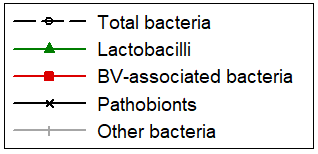 | |

Abbreviations: *BV* bacterial vaginosis, *BV_GV* polybacterial *Gardnerella vaginalis*-containing, *BV_noGV* polybacterial but low *G. vaginalis*, *Conc* concentration, *GV* *G. vaginalis*-dominated, *IQR* inter-quartile range, *LA* lactobacilli and anaerobes, *Li* *L. iners*-dominated, *Lo* other lactobacilli-dominated, *PB* pathobionts-containing, *Pre-tx* pre-treatment visit, *Post-tx* post-treatment visit, *VMB* vaginal microbiota.

**a-f** Figures show changes in VMB characteristics before and after metronidazole treatment, stratified by unusual vaginal discharge symptoms at the pre-treatment visit: Nugent score categories (**a**), bacterial group mean relative abundances (**b**), VMB types (**c**), and median Simpson diversity 1-D (**d**). (**e**) Bacterial group concentrations of participants who did not report unusual vaginal discharge at the pre-treatment visit (n=13; see Table A.4 for 95% confidence intervals). (**f**) Bacterial group concentrations of participants who reported unusual vaginal discharge at the pre-treatment visit (n=55).
