# Supplementary material for: Impact of oral metronidazole treatment on the vaginal microbiota and correlates of treatment failure
Source: Am J Obstet Gynecol. 2020 Feb;222(2):157.e1–157.e13. doi: 10.1016/j.ajog.2019.08.008 (PMC6995998; doi:10.1016/j.ajog.2019.08.008)
Supplement: Appendix B [file mmc2.pdf]

| List of taxa (alphabetical order) | Classification | Phylum/Class (Order) based on NCBI taxonomy browser |
| --- | --- | --- |
| Actinomyces funkei | BV | Actinobacteria/Actinobacteria (Actinomycetales) |
| Actinomyces genus | BV | Actinobacteria/Actinobacteria (Actinomycetales) |
| Actinomyces neuii | BV | Actinobacteria/Actinobacteria (Actinomycetales) |
| Actinomyces urogenitalis | BV | Actinobacteria/Actinobacteria (Actinomycetales) |
| Aerococcus christensenii | BV | Firmicutes/Bacilli (Lactobacillales) |
| Aerococcus genus | BV | Firmicutes/Bacilli (Lactobacillales) |
| Alloprevotella genus | BV | Bacteroidetes/Bacteroidia (Bacteroidales) |
| Anaerococcus genus | BV | Firmicutes/Tissierellia (Tissierellales) |
| Anaerococcus hydrogenalis | BV | Firmicutes/Tissierellia (Tissierellales) |
| Anaerococcus lactolyticus | BV | Firmicutes/Tissierellia (Tissierellales) |
| Anaerococcus murdochii | BV | Firmicutes/Tissierellia (Tissierellales) |
| Anaerococcus obesiensis | BV | Firmicutes/Tissierellia (Tissierellales) |
| Anaerococcus prevotii/tetradius | BV | Firmicutes/Tissierellia (Tissierellales) |
| Arcanobacterium genus | BV | Actinobacteria/Actinobacteria (Actinomycetales) |
| Atopobium deltae | BV | Actinobacteria/Coriobacteria (Coriobacteriales) |
| Atopobium genus | BV | Actinobacteria/Coriobacteria (Coriobacteriales) |
| Atopobium vaginae | BV | Actinobacteria/Coriobacteria (Coriobacteriales) |
| Bacteroidales_S24-7_group family | BV | Bacteroidetes/Bacteroidia (Bacteroidales) |
| Betaproteobacteria class | Other | Betaproteobacteria |
| Bifidobacterium bifidum | Other | Actinobacteria/Actinobacteria (Bifidobacteriales) |
| Bifidobacterium breve | Other | Actinobacteria/Actinobacteria (Bifidobacteriales) |
| Bifidobacterium catenulatum/kashiwanohense/ pseudocatenulatum | Other | Actinobacteria/Actinobacteria (Bifidobacteriales) |
| Bifidobacterium longum | Other | Actinobacteria/Actinobacteria (Bifidobacteriales) |
| Bulleidia genus | BV | Firmicutes/Erysipelotrichi (Erysipelotichales) |
| BVAB TM7 | BV | Unclassified (TM7 division) |
| BVAB1 | BV | Firmicutes/Clostridia (Clostridiales) |
| BVAB2 | BV | Firmicutes/Clostridia (Clostridiales) |
| Campylobacter genus | Pathobionts | Epsilonproteobacteria (Campylobacterales) |
| Campylobacter hominis | Pathobionts | Epsilonproteobacteria (Campylobacterales) |
| Carnobacteriaceae family | Other | Firmicutes/Bacilli (Lactobacillales) |
| Catenibacterium mitsuokai | BV | Firmicutes/Erysipelotrichi (Erysipelotichales) |
| Catonella morbi | BV | Firmicutes/Clostridia (Clostridiales) |
| Chlamydia trachomatis | Pathobionts | Chlamydiae (Chlamydiales) |
| Collinsella aerofaciens | BV | Actinobacteria/Coriobacteria (Coriobacteriales) |
| Coprococcus_3 comes | BV | Firmicutes/Clostridia (Clostridiales) |
| Corynebacteriaceae family | Other | Actinobacteria/Actinobacteria (Corynebacteriales) |
| Corynebacterium atypicum | Other | Actinobacteria/Actinobacteria (Corynebacteriales) |
| Corynebacterium glucuronolyticum | Other | Actinobacteria/Actinobacteria (Corynebacteriales) |
| Corynebacterium jeikeium | Other | Actinobacteria/Actinobacteria (Corynebacteriales) |
| Corynebacterium minutissimum/singulare | Other | Actinobacteria/Actinobacteria (Corynebacteriales) |
| Corynebacterium pyruviciproducens | Other | Actinobacteria/Actinobacteria (Corynebacteriales) |
| Corynebacterium_1 amycolatum/jeikeium/ urealyticum/vitaeruminis | Other | Actinobacteria/Actinobacteria (Corynebacteriales) |
| Corynebacterium_1 aurimucosum | Other | Actinobacteria/Actinobacteria (Corynebacteriales) |

| List of taxa (alphabetical order) | Classification | Phylum/Class (Order) based on NCBI taxonomy browser |
|---|---|---|
| Corynebacterium_1 aurimucosum/minutissimum | Other | Actinobacteria/Actinobacteria (Corynebacteriales) |
| Corynebacterium_1 aurimucosum/pseudogenitalium/ tuberculostearicum | Other | Actinobacteria/Actinobacteria (Corynebacteriales) |
| Corynebacterium_1 aurimucosum/simulans/striatum/ xerosis | Other | Actinobacteria/Actinobacteria (Corynebacteriales) |
| Corynebacterium_1 coyleae | Other | Actinobacteria/Actinobacteria (Corynebacteriales) |
| Corynebacterium_1 genus | Other | Actinobacteria/Actinobacteria (Corynebacteriales) |
| Corynebacterium_1 minutissimum | Other | Actinobacteria/Actinobacteria (Corynebacteriales) |
| Corynebacterium_1 pseudogenitalium | Other | Actinobacteria/Actinobacteria (Corynebacteriales) |
| Corynebacterium_1 riegelii | Other | Actinobacteria/Actinobacteria (Corynebacteriales) |
| Corynebacterium_1 tuscaniense | Other | Actinobacteria/Actinobacteria (Corynebacteriales) |
| Delftia acidovorans/lacustris/tsuruhatensis | Other | Betaproteobacteria (Burkholderiales) |
| Dermabacter hominis | Other | Actinobacteria/Actinobacteria (Micrococcales) |
| Dermabacter jinjuensis | Other | Actinobacteria/Actinobacteria (Micrococcales) |
| Dialister genus | BV | Firmicutes/Negativicutes (Veillonellales) |
| Dialister micraerophilus/microaerophilus | BV | Firmicutes/Negativicutes (Veillonellales) |
| Dialister propionicifaciens | BV | Firmicutes/Negativicutes (Veillonellales) |
| Enterococcus azikeevi/durans/faecalis/faecium/ hirae/lactis/mundtii/raffinosus/ratti/rivorum/thailandicus/villorum | Pathobionts | Firmicutes/Bacilli (Lactobacillales) |
| Enterococcus durans/faecalis/faecium | Pathobionts | Firmicutes/Bacilli (Lactobacillales) |
| Enterococcus faecalis | Pathobionts | Firmicutes/Bacilli (Lactobacillales) |
| Enterococcus faecium | Pathobionts | Firmicutes/Bacilli (Lactobacillales) |
| Escherichia/Shigella genus | Pathobionts | Gammaproteobacteria (Enterobacterales) |
| Ezakiella genus | BV | Firmicutes/Tissierellia (unclassified Tissierellia) |
| Facklamia hominis | BV | Firmicutes/Bacilli (Lactobacillales) |
| Faecalibacterium genus | BV | Firmicutes/Clostridia (Clostridiales) |
| Faecalibacterium prausnitzii | BV | Firmicutes/Clostridia (Clostridiales) |
| Family_XI family | Other | Cyanobacteria (order not described) |
| Family_XIII family | Other | Cyanobacteria (order not described) |
| Family_XIII_UCG-001 genus | Other | Cyanobacteria (order not described) |
| Fastidiosipila genus | BV | Firmicutes/Clostridia (Clostridiales) |
| Fenollaria massiliensis strain DNF00604 | BV | Firmicutes/Clostridia (Clostridiales) |
| Finegoldia genus | BV | Firmicutes/Tissierellia (Tissierellales) |
| Finegoldia magna | BV | Firmicutes/Tissierellia (Tissierellales) |
| Fusobacterium equinum/gonidiaformans | BV | Fusobacteria/Fusobacteriia (Fusobacteriales) |
| Fusobacterium genus | BV | Fusobacteria/Fusobacteriia (Fusobacteriales) |
| Fusobacterium nucleatum | BV | Fusobacteria/Fusobacteriia (Fusobacteriales) |
| Gardnerella genus | BV | Actinobacteria/Actinobacteria (Bifidobacteriales) |
| Gardnerella vaginalis | BV | Actinobacteria/Actinobacteria (Bifidobacteriales) |
| Gemella asaccharolytica | BV | Firmicutes/Bacilli (Bacillales) |
| Gemella genus | BV | Firmicutes/Bacilli (Bacillales) |
| Gemella haemolysans/sanguinis/taiwanensis | BV | Firmicutes/Bacilli (Bacillales) |
| Gemella parahaemolysans | BV | Firmicutes/Bacilli (Bacillales) |
| Granulicatella elegans | BV | Firmicutes/Bacilli (Lactobacillales) |

| List of taxa (alphabetical order) | Classification | Phylum/Class (Order) based on NCBI taxonomy browser |
|---|---|---|
| Granulicatella genus | BV | Firmicutes/Bacilli (Lactobacillales) |
| Haemophilus genus | Pathobionts | Gammaproteobacteria (Pasteurellales) |
| Haemophilus haemolyticus/influenzae | Pathobionts | Gammaproteobacteria (Pasteurellales) |
| Haemophilus haemolyticus/influenzae/quentini | Pathobionts | Gammaproteobacteria (Pasteurellales) |
| Haemophilus influenzae/parainfluenzae | Pathobionts | Gammaproteobacteria (Pasteurellales) |
| Haemophilus parainfluenzae | Pathobionts | Gammaproteobacteria (Pasteurellales) |
| Howardella genus | BV | Firmicutes/Clostridia (Clostridiales) |
| Klebsiella oxytoca/pneumoniae/variicola | Pathobionts | Gammaproteobacteria (Enterobacterales) |
| Lachnospiraceae family | BV | Firmicutes/Clostridia (Clostridiales) |
| Lachnospiraceae_FE2018_group genus | BV | Firmicutes/Clostridia (Clostridiales) |
| Lactobacillus brevis W63 | Lactobacilli | Firmicutes/Bacilli (Lactobacillales) |
| Lactobacillus coleohominis | Lactobacilli | Firmicutes/Bacilli (Lactobacillales) |
| Lactobacillus crispatus/acidophilus/casei/gallinarum | Lactobacilli | Firmicutes/Bacilli (Lactobacillales) |
| Lactobacillus crispatus/gasseri/helveticus/johnsonii/kefiranofaciens | Lactobacilli | Firmicutes/Bacilli (Lactobacillales) |
| Lactobacillus fermentum | Lactobacilli | Firmicutes/Bacilli (Lactobacillales) |
| Lactobacillus fermentum/curieae/delbrueckii/ingluviei/oris/plantarum | Lactobacilli | Firmicutes/Bacilli (Lactobacillales) |
| Lactobacillus fermentum/gasseri/reuteri/vaginalis | Lactobacilli | Firmicutes/Bacilli (Lactobacillales) |
| Lactobacillus fermentum/mucosae | Lactobacilli | Firmicutes/Bacilli (Lactobacillales) |
| Lactobacillus fornicalis/jensenii | Lactobacilli | Firmicutes/Bacilli (Lactobacillales) |
| Lactobacillus gasseri | Lactobacilli | Firmicutes/Bacilli (Lactobacillales) |
| Lactobacillus genus | Lactobacilli | Firmicutes/Bacilli (Lactobacillales) |
| Lactobacillus iatae/johnsonii/taiwanensis | Lactobacilli | Firmicutes/Bacilli (Lactobacillales) |
| Lactobacillus iners | Lactobacilli | Firmicutes/Bacilli (Lactobacillales) |
| Lactobacillus jensenii | Lactobacilli | Firmicutes/Bacilli (Lactobacillales) |
| Lactobacillus johnsonii/prophage/taiwanensis | Lactobacilli | Firmicutes/Bacilli (Lactobacillales) |
| Lactobacillus johnsonii/taiwanensis/gasseri | Lactobacilli | Firmicutes/Bacilli (Lactobacillales) |
| Lactobacillus mucosae | Lactobacilli | Firmicutes/Bacilli (Lactobacillales) |
| Lactobacillus plantarum W21 | Lactobacilli | Firmicutes/Bacilli (Lactobacillales) |
| Lactobacillus reuteri | Lactobacilli | Firmicutes/Bacilli (Lactobacillales) |
| Lactobacillus ruminis | Lactobacilli | Firmicutes/Bacilli (Lactobacillales) |
| Lactobacillus salivarius | Lactobacilli | Firmicutes/Bacilli (Lactobacillales) |
| Lactobacillus vaginalis | Lactobacilli | Firmicutes/Bacilli (Lactobacillales) |
| Lactobacillus vaginalis/reuteri | Lactobacilli | Firmicutes/Bacilli (Lactobacillales) |
| Lawsonella clevelandensis | Other | Actinobacteria/Actinobacteria (Corynebacteriales) |
| Lawsonella genus | Other | Actinobacteria/Actinobacteria (Corynebacteriales) |
| Mageeibacillus indolicus (formerly BVAB3) | BV | Firmicutes/Clostridia (Clostridiales) |
| Megasphaera genus | BV | Firmicutes/Negativicutes (Veillonellales) |
| Micrococcaceae family | Other | Actinobacteria/Actinobacteria (Micrococcales) |
| Mobiluncus curtisii | BV | Actinobacteria/Actinobacteria (Actinomycetales) |
| Mobiluncus genus | BV | Actinobacteria/Actinobacteria (Actinomycetales) |
| Mobiluncus mulieris | BV | Actinobacteria/Actinobacteria (Actinomycetales) |
| Moryella genus | BV | Firmicutes/Clostridia (Clostridiales) |
| Moryella indoligenes | BV | Firmicutes/Clostridia (Clostridiales) |

| List of taxa (alphabetical order) | Classification | Phylum/Class (Order) based on NCBI taxonomy browser |
|---|---|---|
| Murdochiella asaccharolytica | BV | Firmicutes/Tissierellia (Tissierellales) |
| Mycoplasma genus | BV | Tenericutes/Mollicutes (Mycoplasmatales) |
| Mycoplasma hominis | BV | Tenericutes/Mollicutes (Mycoplasmatales) |
| Mycoplasmataceae family | BV | Tenericutes/Mollicutes (Mycoplasmatales) |
| Negativicoccus genus | BV | Firmicutes/Negativicutes (Veillonellales) |
| Neisseria genus | Pathobionts | Betaproteobacteria (Neisseriales) |
| Neisseria gonorrhoeae/meningitidis | Pathobionts | Betaproteobacteria (Neisseriales) |
| Olsenella genus | BV | Actinobacteria/Coriobacteriia (Coriobacteriales) |
| Paeniglutamicibacter genus | Other | Actinobacteria/Actinobacteria (Micrococcales) |
| Parvimonas genus | BV | Firmicutes/Tissierellia (Tissierellales) |
| Parvimonas micra | BV | Firmicutes/Tissierellia (Tissierellales) |
| Peptococcus genus | BV | Firmicutes/Clostridia (Clostridiales) |
| Peptoniphilus asaccharolyticus/grossensis/harei | BV | Firmicutes/Tissierellia (Tissierellales) |
| Peptoniphilus coxii | BV | Firmicutes/Tissierellia (Tissierellales) |
| Peptoniphilus duerdenii | BV | Firmicutes/Tissierellia (Tissierellales) |
| Peptoniphilus genus | BV | Firmicutes/Tissierellia (Tissierellales) |
| Peptoniphilus gorbachii/rhinitidis | BV | Firmicutes/Tissierellia (Tissierellales) |
| Peptoniphilus lacrimalis | BV | Firmicutes/Tissierellia (Tissierellales) |
| Peptoniphilus massiliensis | BV | Firmicutes/Tissierellia (Tissierellales) |
| Peptostreptococcaceae family | BV | Firmicutes/Clostridia (Clostridiales) |
| Peptostreptococcus anaerobius | BV | Firmicutes/Clostridia (Clostridiales) |
| Peptostreptococcus genus | BV | Firmicutes/Clostridia (Clostridiales) |
| Phascolarctobacterium genus | BV | Firmicutes/Negativicutes (Acidaminococcales) |
| Porphyromonas genus | BV | Bacteroidetes/Bacteroidia (Bacteroidales) |
| Porphyromonas uenonis | BV | Bacteroidetes/Bacteroidia (Bacteroidales) |
| Prevotella amnii | BV | Bacteroidetes/Bacteroidia (Bacteroidales) |
| Prevotella bivia | BV | Bacteroidetes/Bacteroidia (Bacteroidales) |
| Prevotella bivia/denticola | BV | Bacteroidetes/Bacteroidia (Bacteroidales) |
| Prevotella buccalis | BV | Bacteroidetes/Bacteroidia (Bacteroidales) |
| Prevotella disiens | BV | Bacteroidetes/Bacteroidia (Bacteroidales) |
| Prevotella genus | BV | Bacteroidetes/Bacteroidia (Bacteroidales) |
| Prevotella intermedia | BV | Bacteroidetes/Bacteroidia (Bacteroidales) |
| Prevotella melaninogenica | BV | Bacteroidetes/Bacteroidia (Bacteroidales) |
| Prevotella timonensis | BV | Bacteroidetes/Bacteroidia (Bacteroidales) |
| Prevotella_6 corporis | BV | Bacteroidetes/Bacteroidia (Bacteroidales) |
| Prevotellaceae family | BV | Bacteroidetes/Bacteroidia (Bacteroidales) |
| Propionimicrobium genus | Other | Actinobacteria/Actinobacteria (Propionibacteriales) |
| Prevotella colorans | BV | Bacteroidetes/Bacteroidia (Bacteroidales) |
| Proteobacteria phylum | Other | Proteobacteria |
| Raoultella genus | Pathobionts | Gammaproteobacteria (Enterobacterales) |
| Rickettsiales_Incertae_Sedis family | Pathobionts | Alphaproteobacteria (Rickettsiales) |
| Rikenellaceae_RC9_gut_group genus | BV | Bacteroidetes/Bacteroidia (Bacteroidales) |
| Roseburia genus | BV | Firmicutes/Clostridia (Clostridiales) |
| Ruminococcaceae family | BV | Firmicutes/Clostridia (Clostridiales) |

| List of taxa (alphabetical order) | Classification | Phylum/Class (Order) based on NCBI taxonomy browser |
|---|---|---|
| Ruminococcaceae_UCG-014 genus | BV | Firmicutes/Clostridia (Clostridiales) |
| Saccharibacteria phylum | BV | Unclassified (TM7 division) |
| Senegalimassilia genus | BV | Actinobacteria/Coriobacteriia (Coriobacteriales) |
| Shuttleworthia genus | BV | Firmicutes/Clostridia (Clostridiales) |
| Slackia exigua | BV | Actinobacteria/Coriobacteriia (Eggerthellales) |
| Sneathia amnii | BV | Fusobacteria/Fusobacteriia (Fusobacteriales) |
| Sneathia amnii/sanguinegens | BV | Fusobacteria/Fusobacteriia (Fusobacteriales) |
| Sneathia genus | BV | Fusobacteria/Fusobacteriia (Fusobacteriales) |
| Sneathia sanguinegens | BV | Fusobacteria/Fusobacteriia (Fusobacteriales) |
| SR1_(Absconditabacteria) phylum | BV | Unclassified (Absconditabacteria division) |
| Staphylococcus | Pathobionts | Firmicutes/Bacilli (Bacillales) |
| Staphylococcus aureus/devriesei/epidermidis/haemolyticus | Pathobionts | Firmicutes/Bacilli (Bacillales) |
| Staphylococcus epidermidis/haemolyticus | Pathobionts | Firmicutes/Bacilli (Bacillales) |
| Staphylococcus epidermidis/haemolyticus/hominis | Pathobionts | Firmicutes/Bacilli (Bacillales) |
| Staphylococcus genus | Pathobionts | Firmicutes/Bacilli (Bacillales) |
| Staphylococcus haemolyticus | Pathobionts | Firmicutes/Bacilli (Bacillales) |
| Staphylococcus haemolyticus/petrasii | Pathobionts | Firmicutes/Bacilli (Bacillales) |
| Streptococcus agalactiae/pyogenes | Pathobionts | Firmicutes/Bacilli (Lactobacillales) |
| Streptococcus alactolyticus/equinus/gallolyticus/ macedonicus/pasteuri/pasteurianus | Pathobionts | Firmicutes/Bacilli (Lactobacillales) |
| Streptococcus anginosus/milleri | Pathobionts | Firmicutes/Bacilli (Lactobacillales) |
| Streptococcus dentisani/infantis/mitis/ oligofermentans/oralis/pneumoniae/ pseudopneumoniae/sanguinis/tigurinus | Pathobionts | Firmicutes/Bacilli (Lactobacillales) |
| Streptococcus equinus/infantarius/lutetiensis | Pathobionts | Firmicutes/Bacilli (Lactobacillales) |
| Streptococcus genus | Pathobionts | Firmicutes/Bacilli (Lactobacillales) |
| Sutterella genus | Other | Betaproteobacteria (Burkholderiales) |
| Sutterella morbirenis/sanguinus | Other | Betaproteobacteria (Burkholderiales) |
| Ureaplasma genus | BV | Tenericutes/Mollicutes (Mycoplasmatales) |
| Ureaplasma parvum/urealyticum | BV | Tenericutes/Mollicutes (Mycoplasmatales) |
| Ureaplasma urealyticum | BV | Tenericutes/Mollicutes (Mycoplasmatales) |
| Varibaculum cambriense | BV | Actinobacteria/Actinobacteria (Actinomycetales) |
| Varibaculum genus | BV | Actinobacteria/Actinobacteria (Actinomycetales) |
| Veillonella dispar | BV | Firmicutes/Negativicutes (Veillonellales) |
| Veillonella genus | BV | Firmicutes/Negativicutes (Veillonellales) |
| Veillonella montpellierensis | BV | Firmicutes/Negativicutes (Veillonellales) |
